# Expression, localization and regulation of NADPH oxidases in pancreatic beta cells

**DOI:** 10.1080/13510002.2025.2568300

**Published:** 2025-10-06

**Authors:** Davidson Correa de Almeida, Eloisa Aparecida Vilas-Boas, Paulo Henrique Coelho Ferreira, Sandra Mara Ferreira, Angelo Rafael Carpinelli, Fernanda Ortis

**Affiliations:** a Laboratory of Study of β Cell Death Molecular Mechanisms, Department of Cell and Development Biology, Institute of Biomedical Sciences, University of São Paulo, São Paulo, Brazil; b Laboratory of Endocrine Pathophysiology and Redox Processes, Department of Clinical and Toxicological Analysis, School of Pharmaceutical Sciences, University of São Paulo, São Paulo, Brazil; c Laboratory of the Endocrine Pancreas and Metabolism, Department of Structural and Functional Biology, Institute of Biology, State University of Campinas, Campinas, Brazil; d Laboratory of Pancreatic Cellular and Biomaterials Engineering, J. Crayton Pruitt Family Department of Biomedical Engineering, Herbert Wertheim College of Engineering, University of Florida, Gainesville, FL, United States of America; e Laboratory of Physiology of Insulin Secretion, Department of Physiology and Biophysics, Institute of Biomedical Sciences, University of São Paulo, São Paulo, Brazil; f Instituto Nacional de Ciência e Tecnologia de Bioanalítica (INCTBio), Campinas, Brazil

**Keywords:** NADPH oxidases, pancreatic beta cells, insulin secretion, insulitis, diabetes

## Abstract

**Objectives:**

Reactive oxygen species (ROS) are short-lived and act in a site-specific manner, underscoring the importance of identifying the subcellular localization of their sources. ROS-generating NADPH oxidases (NOX) regulate pancreatic beta cell (dys)function. However, their subcellular localization and cytokine-mediated regulation in these cells remain largely unknown. We characterized the expression, subcellular localization and time-dependent cytokine-induced regulation of NOX isoforms in beta cells.

**Methods:**

Isoforms were studied via RT-qPCR, immunoblotting and immunofluorescence in rat islets and beta cell lines.

**Results:**

Beta cells express DUOX1 and DUOX2 proteins and *Duoxa2* transcripts; lacking *Duoxa1* expression. In INS-1E cells, NOX1 and DUOX1 localize in the endoplasmic reticulum (ER); DUOX2 in insulin vesicles; and NOX2 and NOX4 in vesicles, ER and plasma membrane. In INS-1E, cytokines increased expression of *Nox1* and *Duox1* at 4-8 h (returning to baseline at 16 h) and *Nox2* and *p47phox* at 8 h (persisting until 24 h). *Duox(a)2*, *p67phox* and *p40phox* were downregulated and DUOX1 upregulated at 16-24 h.

**Conclusion:**

The absence of *Duoxa1* in beta cells might lead to DUOX1 mismatching, impairing its trafficking and activity. NOXs in beta cells are diverse in subcellular localization and cytokine-induced regulation, suggesting their isoform-specific involvement in beta cell function, stress and apoptosis.

## Introduction

Reactive oxygen species (ROS) mediate redox signaling, regulating diverse cellular processes through modulation of membrane receptors, kinases, phosphatases, transcription factors, ion channels and other molecular targets. However, dysregulated ROS production disrupts redox signaling, leading to oxidative stress and accumulated oxidative damage to proteins, lipids and DNA [1–3]. NADPH oxidases (NOX) are transmembrane flavoprotein cytochromes. As *professional* oxidases, their sole enzymatic function is the generation of ROS – superoxide ($O_{2}^{∙-}$) or hydrogen peroxide (H_2_O_2_) – from O_2_ [4–7]. The mammalian NOX family has seven homologs: NOX1-4; NOX5 (absent in rodents) and dual oxidase (DUOX) 1 and 2 [4–7]. DUOX are distinguished by the presence of a peroxidase-homology ectodomain – hence the name *dual* oxidase, although the functional relevance of this domain remains debated [8].

To form functional holoenzymes, NOXs associate with transmembrane *scaffolding partners*, cytosolic *regulatory factors* and Rac GTPases [4,8]. Overall, NOX isoenzymes are structurally heterogenous; differing in their subunit compositions, modes of activation and ROS product [4,8]. NOX1-4 bind the scaffolding partner p22phox [4,8], while DUOX1 and DUOX2 bind the scaffolding partners DUOX Activator (DUOXA) 1 or 2 [7,9]. DUOXAs are essential for the proper maturation, assembly, trafficking and enzymatic activity of DUOXs [10–12]. NOX1 and NOX2 require the stimulus-dependent recruitment of Rac and cytosolic factors: NOX1 binds NOXA1 and NOXO1; NOX2 binds p67phox, p47phox and p40phox. In contrast, NOX3 and NOX4 are constitutively active: NOX3 via constitutive binding to NOXO1, while NOX4 functions independently of Rac and recruitment of regulatory factors. NOX5 and DUOXs are activated upon binding of free cytosolic Ca^2+^. Finally, NOX1-3 and NOX5 generate $O_{2}^{∙-}$ and NOX4 and DUOXs generate H_2_O_2_ [4,8]. NOXs are also diverse in their intracellular localization and tissue expression [4,7,13,14]; participating in various physiological functions (including microbial killing and host defence, vasoregulation and angiogenesis, hormone synthesis, cell differentiation, proliferation and migration) and in a gamut of pathologies (such as chronic inflammation, cardiomyopathy and hypertension, neurodegeneration, cancer) [4,7].

Pancreatic β cells constitutively express NOX1, NOX2 and NOX4, alongside all their respective subunits. In contrast, NOX3 and NOX5 are not expressed in these cells [15–19]. Reflecting the *dual* role of ROS in other cell types, NOXs are involved in both physiological and pathological events in β cells [20–23]. ROS production and NOX expression in β cells are stimulated by glucose [24–28], fatty acids [26,29] and proinflammatory cytokines [26,30,31]. Notably, exogenous ROS can stimulate insulin secretion even in the absence of metabolic stimuli [25,28]. General antioxidants and global NOX inhibition with DPI (a non-specific flavoenzyme inhibitor) decrease glucose-stimulated insulin secretion (GSIS) and impair glucose-stimulated Ca^2+^ signaling [25,32,33]. Conversely, these agents also mitigate GSIS dysfunction induced by cytokines [34] and fatty acids [35].

However, the isoform-specific role of NOX in β cells remains elusive and functional studies yielded seemingly contradictory findings. For instance, knockout (KO) of *Nox2* and, to a lesser extent, of *Nox4* enhanced GSIS in mouse islets [18]. On the other hand, knockdown (KD) of *p47phox* (NOX2 regulatory factor) in rat islets, *Nox4* KO in mouse islets and *Nox4* KD in rat INS-1E cells decreased GSIS [33,36]. These reports suggest that NOX regulation of GSIS might be bidirectional (stimulatory and inhibitory) and isoform-specific. Additionally, NOX1 inhibition prevents cytokine-induced GSIS dysfunction and β cell death [31]. Similarly, *Nox2* KO or KD alleviate the effects of cytokines, glucotoxicity and lipotoxicity [37–39]. *Nox2* KO also prevents streptozotocin-induced Diabetes [40]. NOX4 inhibitors protect β cells against cytokines and glucolipotoxicity [41–43] and *Nox4* KO mitigates inflammation caused by high-fat diet [44]. Altogether, these findings indicate the involvement of multiple NOX isoforms in both β cell function and dysfunction.

The major challenges in researching NOX expression, their activity and regulation are the typically very low expression levels [18,30,45], the limited specificity of commercially-available antibodies [18,45] and non-selectivity of pharmacological inhibitors [41,46] and redox-sensitive probes [47,48]. Accordingly, many aspects of NOX biology in β cells remain unresolved [23]. To our knowledge, expression of DUOX1 and DUOX2 in β cells was never formally investigated. DUOXs are the only NOX isoforms combining Ca^2+^ activation (feature shared only with NOX5, absent in β cells) with generation of H_2_O_2_ instead of $O_{2}^{∙-}$ (feature shared only with NOX4, constitutively active) [6,9,49]. DUOX activity is regulated by NADPH and Ca^2+^: elevated NADPH levels reduce their sensitivity to Ca^2+^ [50] and prolonged exposure to Ca^2+^ gradually decelerates their catalytic rate [51]. Consequently, DUOX activity is restricted to transient bursts of H_2_O_2_ generation localized at microdomains of high [Ca^2+^]_i_ (*hotspots*) [50,51]. Therefore, DUOXs might link redox signaling to Ca^2+^ signaling in β cells. Also, the intracellular localization of NOX isoforms in β cells is mostly unknown, aside for reports of NOX2 colocalization with insulin vesicles and lysosomes [18] and the widespread cytoplasmic distribution of NOX2 and NOX4 observed in human β cells [41]. Since redox signaling is highly spatially-dependent [14,52], determining the intracellular localization of NOXs in β cells is essential for understanding their function. Finally, regulation of some NOX components by proinflammatory cytokines might be time-dependent [26,30,38]. However, characterization of this phenomenon across the entire NOX repertoire remains lacking.

Thus, our aim was to investigate in β cells (i) the expression of DUOX(A) (DUOX and DUOXA) 1 and 2; (ii) the intracellular localization of NOX isoforms; and (iii) the time-dependent regulation of NOX expression by proinflammatory cytokines. We show that isolated rat pancreatic islets and the two rat β cell lines INS-1E and BRIN-BD11 express *Duox1*, *Duox2* and *Duoxa2* transcripts, DUOX1 and DUOX2 proteins and lack *Duoxa1* transcripts. In INS-1E, NOX1 and DUOX1 are expressed in the endoplasmic reticulum (ER), DUOX2 in insulin vesicles and NOX2 and NOX4 in insulin vesicles, ER and plasma membrane. No isoforms showed significant nuclear localization. In INS-1E, cytokines increased mRNA expression of *Nox1*, *Duox1* (early and transient effects), *Nox2* and *p47phox* (early and sustained effects); decreased mRNA expression of *Duox(a)2*, *p67phox* and *p40phox*; and increased protein expression of DUOX1 (late effects).

## Material and methods

### Material

All reagents, primers and antibodies are described in Additional File 1: Material. Hank’s Balanced Salt Solution (HBSS): NaCl 140 mM, KCl 5 mM, MgSO_4_⋅7H_2_O 0.8 mM, Na_2_PO_4_⋅12H_2_O 0.3 mM, KH_2_PO_4_ 0.4 mM, CaCl_2_⋅2H_2_O 1 mM, NaHCO_3_ 4 mM, glucose 10 mM, 0.2% BSA, pH 7.4. TAE buffer: Tris base 40 mM, acetic acid 20 mM, EDTA 1 mM, pH 8.2. TBE buffer: Tris base 89 mM, boric acid 89 mM, EDTA 2 mM, pH 8.2. TBST buffer: Tris base 20 mM, NaCl 150 mM, 0.1% Tween 20. PBST buffer: 0.1% Tween 20, pH 7.4 in PBS. Loading buffer: 0.04% bromophenol blue, 6.7% sucrose in TAE. RIPA buffer: NaCl 150 mM, 1% Triton X-100, 0.5% sodium deoxycholate, 0.1% SDS, Tris 50 mM, pH 8.0. Laemmli buffer: 2% SDS, 5% 2-mercaptoethanol, 10% glycerol, 0.005% bromophenol blue, Tris-HCl 62.5 mM, pH 6.8. Ponceau S solution: 0.1% Ponceau S, 5% acetic acid. Fixing buffer: 4% paraformaldehyde, pH 6.9 in PBS. Antigen recuperation buffer: citric acid 10 mM, 0.05% Tween 20, pH 6.0. Permeabilization buffer: 0.2% Triton X-100 in TBS (immunohistochemistry) or 0.5% Triton X-100 in PBS (for immunocytochemistry). Blocking buffer: 5% non-fat dry milk in TBST (for immunoblotting), 5% BSA in TBST (for immunohistochemistry) or in PBST (for immunocytochemistry). Antibody buffer: 5% BSA in TBST (for immunoblotting) or 1% BSA in TBST (for immunohistochemistry) or in PBST (for immunocytochemistry).

### Isolation of pancreatic islets

Male Wistar rats (RRID:RGD_13508588) were maintained at 24 ± 2°C with water and food *ad libitum* in light-dark cycles of 12 h-12 h until 60–90 days of age. Animals were euthanized and pancreata were either dissected on ice and immediately used for immunohistochemistry or perfused through the common bile duct with 0.07% collagenase type V in HBSS and digested (37°C/25 min) [53]. 98–670 islets/animal were individually collected in HBSS on ice with micropipettes under a stereoscope, washed 2× in PBS and immediately used for RT-qPCR and immunoblotting. Lungs and kidneys were collected on ice, immediately frozen in liquid N_2_ and stored at –70°C for RT-qPCR or immunoblotting. Lungs and kidneys were used as controls for high and low *Duox(a)* gene and protein expression, respectively (NCBI GenBank ID: 266807, 79107, 311374 and 499879) [4,7]. All animal procedures were approved by the respective ethics committee (CEUA/ICB-USP), certificates 66/2015/CEUA (declarations CEUA.3.2018 and CEUA.9.2019) and 66/2016/CEUA (declaration CEUA.59.2018).

### Cell culture and treatment

Insulin-secreting INS-1E cells (RRID:CVCL_0351) were seeded (4–8 × 10^4^ cells/cm^2^ in 2 cm^2^ or 10 cm^2^ wells or 25 cm^2^ flasks) and cultured (37°C, 5% CO_2_) in RPMI 1640 medium (with HEPES 10 mM, sodium pyruvate 1 mM, penicillin 10 U/mL, streptomycin 10 μg/mL, sodium bicarbonate 2 g/L, 2-mercaptoethanol 50 μM and 5% inactivated FBS) [54] for at least 48 h before experiments. Cells were cultured in TC-treated polystyrene plates (for RT-qPCR and immunoblotting) or poli-L-lysine-treated coverslips (for immunocytochemistry).

Insulin-secreting BRIN-BD11 cells (RRID:CVCL_6811) were seeded (2–4 × 10^4^ cells/cm^2^ in 25 cm^2^ flasks) and cultured in TC-treated polystyrene plates (37°C, 5% CO_2_) in RPMI 1640 medium (with penicillin 10 U/mL, streptomycin 10 μg/mL, sodium bicarbonate 2 g/L and 10% inactivated FBS) [55] for at least 24 h before experiments.

Total protein and RNA extracts from the rat thyroid cell line PCCL3 (RRID:CVCL_6712) were used as a control for high *Duox(a)* gene and protein expression [4,7].

#### Cell treatment

INS-1E were treated with thapsigargin 1 μM for 16 h or with IL-1β 10 U/mL + IFNγ 14 U/mL + TNF 100 U/mL for 1, 2, 4, 8, 16 and 24 h or left untreated for 24 h. Thapsigargin treatment was used as a positive control for induction of ER stress and cell death [56]. The combination and concentration of cytokines used are sufficient to induce β cell death, thus mimicking the proinflammatory environment during Type 1 Diabetes *mellitus* (T1DM) [57–59].

### RT-qPCR

Frozen tissues (50–100 mg/sample) were homogenized in TRIzol Reagent at 4°C. Isolated islets (98–670 islets/animal) and β cell lines (seeded at 2–8 × 10^4^ cells/cm^2^) were resuspended in TRIzol Reagent at 4°C. Total RNA was extracted from lysates with RNase-free glycogen, following the manufacturer’s instructions. Concentration and absorbance in 280, 260 and 230 nm from extracts were measured in NanoDrop 2000 to assess purity (data not shown). Total RNA was fractionated by gel electrophoresis (described below) to determine integrity (data not shown). Only non-contaminated non-degraded extracts were used.

RNA was treated with ezDNase (37°C/20 min) and transcribed to cDNA with SuperScript IV VILO Master Mix, following the manufacturer’s instructions, in a SimpliAmp thermal cycler (25°C/10 min, 50°C/50 min, 85°C/5 min). Within each experiment, the same amount of RNA was used for cDNA synthesis for all samples. In experiments investigating *Duox(a)* expression in tissues and cell lines, 4 μg/reaction was used (except for PCCL3, where 2 μg/reaction was used). In cytokine treatment experiments, 3.5–5.0 μg/reaction was used. No amplification control (NAC) reactions (omitting reverse transcriptase during cDNA synthesis) were prepared as controls for gDNA amplification.

qPCR was performed with 300 nM forward/reverse specific primers and PowerUp SYBR Green Master Mix in 10 μL reactions in technical triplicates in Rotor-Gene 6000 or Rotor-Gene Q thermal cyclers, following the manufacturer’s instructions. The same amount of cDNA was used for all samples: 100 ng/reaction for tissues and for cytokine treatment experiments; or 10 ng/reaction for cell lines. No Template Control (NTC) reactions were prepared in triplicates in each qPCR run to control for unspecific amplification. Fast Cycling Mode was used (UDG activation: 50°C/2 min, Dual-lock DNA polymerase activation: 95°C/2 min, 40 cycles: 95°C/3 seg → 60°C/30 seg, final denaturation: 95°C/15 seg, dissociation: 60 → 95°C + 1°C/step 5 seg/step) with Auto-Gain Optimization = 1–5 FI, Dynamic Tube and Slop Correct active and Threshold = 0.05 dRn.

Expression of reference genes was analysed with the 2^-CT^/2^-CT^ method, without significant variation between groups (analysis not shown). Expression of genes of interest was analysed with the 2^-ΔCT^ or the 2^-ΔΔCT^ method, calibrated by the respective reference gene and control group, as indicated [60]. The absence of amplification in NTC and NAC reactions was confirmed by analysis of dissociation curves for all samples and, occasionally, gel electrophoresis (described below). Genes analysed: *Gapdh* and *Rn18s* (reference genes, as indicated); *Aft3*, *Chop* (ER stress markers) and *Cxcl10* (cytokine-induced chemokine); *Nox1*, *Nox2*, *Nox4*, *Duox1* and *Duox2* (NOX isoforms); *p22phox* (NOX1–4 partner); *Duoxa1* and *Duoxa2* (DUOX partners); *p67phox*, *p47phox* and *p40phox* (NOX2 factors); *Noxa1* and *Noxo1* (NOX1/3 factors); and *Rac1* (NOX1–2 factor) – thus encompassing the entire NOX repertoire available in β cells. RT-qPCR data are presented in Additional File 2: Dissociation Curve, in Additional File 3: DUOX Expression and in Additional File 6: Treatment.

#### RNA and DNA gel electrophoresis

Electrophoresis was performed in 1% agarose gels in TAE buffer for total RNA (0.5–1.0 μg/well) or in 3–4% agarose gels in TBE buffer for qPCR products (entire 10 μL reactions loaded onto wells). Samples were mixed in Loading buffer or Gel Loading Dye Purple 6X immediately before electrophoresis. Gels were pre-stained with GelRed 0.5X and photographed in ImageQuant LAS or E-Gel Imager System. Images were analysed in the ImageLab software. RNA integrity was analysed by measuring 28S/18S signal intensity (data not shown). The apparent molecular weight of qPCR products was estimated by point-to-point semi-log regression. Gels shown are cropped for clarity. Full-length gels are presented in Additional File 7: Supplementary Figures.

### Immunoblotting

Frozen tissues (15–25 mg/sample) were homogenized in ice-cold RIPA buffer with Halt Protease Inhibitor Cocktail. Total protein extracts from isolated islets were purified from lysates in TRIzol Reagent, following the manufacturer’s instructions and suspended in 10% SDS. INS-1E and BRIN-BD11 cells were washed 2× in ice-cold PBS and lysed in RIPA buffer with Halt Protease Inhibitor Cocktail (4°C/5 min). Lysates were clarified (14000 g/15 min at 4°C). Protein concentration was estimated with the BCA Protein Assay Kit, following the manufacturer’s instructions, in the BioTek Synergy H1 spectrometer. Samples in Laemmli buffer were fractionated by SDS-PAGE and transferred to 0.2 μm nitrocellulose membranes. Within each experiment, the same amount of protein was loaded for all samples (15–20 μg/well). Boiling samples before electrophoresis was avoided to minimize NOX4 proteolysis [45]. Membranes were stained with Ponceau S (5 min) and photographed in ImageQuant LAS [61,62]. Membranes were then destained, incubated in blocking buffer (1 h), in antibody buffer with primary antibodies (4°C overnight), in blocking buffer with secondary antibodies (1 h), in Clarity Western ECL Substrate (10 seg-5 min) and photographed in ImageQuant LAS. The apparent molecular weight of proteins was estimated by point-to-point semi-log regression in the ImageLab software. Signal intensity was normalized by the full-lane total protein Ponceau S staining and the respective control group, as indicated [61]. Signal quantification values (Ponceau S and proteins of interest) are presented in Additional File 3: DUOX Expression and in Additional File 6: Treatment. Blots shown are cropped for clarity. Full-length blots are presented in Additional File 7: Supplementary Figures.

### Immunofluorescence

#### Immunohistochemistry (IHC)

Dissected pancreata were washed 2× in ice-cold PBS, incubated in fixing buffer (4°C/21 h), dehydrated in sequential ethanol baths (75%, 95% and 100%), diaphanized 2× in 100% xylene and embedded in molten paraffin wax. Paraffin blocks were sectioned in 6 μm slices in a microtome. Slices placed in poly-L-lysine-coated glass slides dried in an oven (40°C overnight and then 60°C/2 h). Sections were deparaffinized in 100% xylene (2× 12 min), rehydrated in sequential ethanol baths (100%, 95%, 85%, 75%, 2× 5 min each) and washed in dH2O (3× 5 min). Sections were then incubated in antigen recuperation buffer (98°C/20 min), in permeabilization buffer (30 min), in blocking buffer (2 h), in antibody buffer with/without rabbit and Alexa Fluor 488-conjugated mouse primary antibodies (4°C overnight in the dark) and in antibody buffer with/without goat secondary antibodies conjugated with Alexa Fluor 568 (2 h in the dark). Slides were mounted to glass coverslips with ProLong Glass Antifade Mountant with NucBlue Stain (Hoechst 33342) and left to cure (48 h in the dark). DUOX isoforms were indirectly stained with Alexa Fluor 568, insulin directly stained with Alexa Fluor 488 and DNA stained with Hoechst 33342. Controls were incubated either without primary antibodies or without primary antibodies nor secondary antibodies, without significant staining in both controls.

#### Immunocytochemistry (ICC)

Coverslip-cultured INS-1E were washed 2× in PBS, incubated in fixing buffer (10 min), permeabilization buffer (15 min) and blocking buffer (1–2 h). Coverslips were then incubated in antibody buffer with/without rabbit and/or mouse primary antibodies (4°C overnight in the dark), in antibody buffer with/without goat secondary antibodies conjugated with Alexa Fluor 568 or with Alexa Fluor 488 or phalloidin-Alexa Fluor 488 (2 h in the dark), mounted on glass slides with ProLong Glass Antifade Mountant with NucBlue Stain (Hoechst 33342) and left to cure (24–48 h in the dark). NOX isoforms were indirectly stained with Alexa Fluor 568. Insulin, protein disulfide-isomerase (PDI), syntaxin 1, F-actin and lysosomal-associated membrane protein 1 (LAMP1) were stained with Alexa Fluor 488 and DNA was stained with Hoechst 33342 for simultaneous labeling of insulin vesicles, ER, plasma membrane, actin cytoskeleton, lysosomes and nuclei, respectively. Controls were incubated either without primary antibodies or without primary antibodies nor secondary antibodies, without significant staining in both controls.

#### Widefield fluorescent microscopy

Slides were photographed with an Axiocam 702 mono camera mounted in a Zeiss Axio Observer 7 inverted microscope with a LP Plan-Neofluar 20×/0.4 Korr Ph 2 objective (for IHC) or a Plan-Apochromat 100× 1.4 NA oil objective (for ICC) and a HXP 120 V light source. Images were captured as 14-bit 3-channel Z-stacks of 1920 × 1216 × 11 px in 0.293 × 0.293 × 1.940 μm/px scaling (for IHC) or 1920 × 1216 × 20–30 px in 0.059 × 0.059 × 0.240 μm/px scaling (for ICC). DAPI (Ex. 370–410 nm/Em. 430–470 nm), FITC (Ex. 450–490 nm/Em. 500–550 nm) and Rhodamine (Ex. 538–562 nm/Em. 570–640 nm) filters were used, avoiding signal saturation whenever possible. For IHC, rhodamine channels were captured under constant exposure of 3.0 seg.

#### Confocal laser scanning microscopy (CLSM)

Slides were photographed with HyD S detectors in a Leica Stellaris confocal laser scanning microscope (90.1 μm pinhole) with a HC PL Apo CS2 63×/1.4 NA Oil objective and 3× optical zoom and a white light laser (WLL) combined with a Acousto-Optical Beam Splitter (AOBS). Slides were unidirectionally scanned at 700 Hz (0.488 μs/px) and images were captured as 14-bit 3-channel Z-stacks of 1576 × 1576 × 12–24 px in 0.039 × 0.039 × 0.028 μm/px scaling. Channels were captured using spectral positions 420–495 nm/2.5 gain, 504–582 nm/2.5 gain and 584–698 nm/3.3 gain for Hoechst 33342, Alexa Fluor 488 and Alexa Fluor 568, respectively.

#### Staining quantification

Non-deconvoluted subsets of representative planes of 1195 × 1195 px (350 × 350 μm, centered around one islet) and 341 × 341 px (20 × 20 μm, centered around at least one cell) were selected from IHC and ICC widefield images, respectively. Subsets were processed and analysed in the FIJI software: the background was subtracted by a sliding paraboloid of 5 px radius, without obscuring relevant structures. Regions of Interest (ROI) around the insulin-positive area (for IHC) and around cells (for ICC) were automatically drawn with Huang’s thresholding algorithm. The mean ± SD px intensity and calibrated area inside and outside ROIs were measured. For IHC, gaps inside the insulin-positive area were also measured and used as background for further calculations. Integrated density = ROI mean px intensity × ROI calibrated area. Corrected total cell fluorescence (CTCF) = integrated density – (background mean px intensity × ROI calibrated area).

To facilitate visualization, histograms of red channels are displayed with linear contrast-stretching from 500 to 5.000 intensity (for IHC) or from 0 to 8.196 intensity (for ICC). Histograms of blue and green channels are displayed with linear contrast-stretching from 0.1% of the darkest px to 0.1% of the brightest px. The FIJI macros used are shown in Additional File 1: Material. Immunofluorescence quantification data is shown in Additional File 4: px Intensity.

#### Image processing and association analysis

For association analyses, Z-stacks were deconvoluted using the default Fast Interactive algorithm in the ZEN blue software (for widefield images) or using adaptive Lightning deconvolution in the LAS X software (for CLSM images). Subsets of 273 × 273 × 10 px (16 × 16 × 2.4 μm) and 640 × 640 × 11 px (25 × 25 × 2.8 μm) were selected from widefield and CLSM images, respectively, minimizing the inclusion of extracellular unlabeled regions. This volume was sufficient to include at least 1–2 cells. Subsets were processed in the FIJI software: px values were averaged by a 3 × 3 px grid to improve signal-to-noise ratio (only for widefield images), the background was subtracted by a sliding paraboloid of 5 px radius and histograms were contrast-stretched linearly to saturate 0.1% of the brightest px in the whole volume [63]. Such adjustments did not obscure relevant structures. ROIs around cells were automatically drawn with Huang’s thresholding algorithm in FIJI.

Channels were compared with the JACoP (BIOP version) plugin in FIJI [64]. Correlation between any given two fluorophores was measured with Pearson’s correlation coefficient (PCC) and colocalization between any given two fluorophores was measured with Manders’ colocalization coefficients M1 and M2 [63,64]. For colocalization analyses, Otsu’s thresholding algorithm was used. PCC ranges from –1.00 to +1.00 and can be classified as (very) weak (0.00 ≤ |r| ≤ 0.40), moderate (0.40 < |r| ≤ 0.60), or (very) strong (0.60 < |r| ≤ 1.00). M1 and M2 range from 0.00 to 1.00. M1 indicates the fraction of channel A (Alexa Fluor 568 or Alexa Fluor 488) within channel B (Alexa Fluor 488 or Hoechst 33342). Conversely, M2 indicates the fraction of channel B within channel A [63–65].

Representative subsets from each group are displayed as maximum-intensity projections in false-color composites with linear contrast-stretching of histograms from 0.1% of the darkest px to 0.1% of the brightest px. Blue: Hoechst 33342, green: Alexa Fluor 488, red: Alexa Fluor 568. The FIJI macros used are shown in Additional File 1: Material. Immunocytochemistry association data is shown in Additional File 5: Association.

### Data analysis

All experiments were performed in at least 3 biological replicates, except for immunofluorescence experiments. For IHC, 18 islets from 5 pancreata (6 islets each) were analysed. For ICC, 8–12 subsets from 2–3 biological replicates (4 subsets each) for widefield or 10 subsets from 3 biological replicates (3–4 subsets each) for CLSM were analysed. qPCR data were log_10_-transformed and analysed and shown as such. Parameters are shown as mean ± SD or Min-Max box plots, as indicated. Differences between groups were calculated with multiple unpaired t tests followed by Holm-Šídák’s test, ordinary one-way or two-way ANOVA, repeated measures (RM) one-way ANOVA or mixed-effects analysis followed by Tukey’s or Dunnett’s test, as indicated. For Tukey’s test, differences between groups are indicated by Compact Letter Display. Sphericity of data was assumed. For fluorophore associations, groups were also compared with two-tailed one-sample t tests against a hypothetical mean of r = 0 (random distribution without association, Additional File 5: Association) [66]. Statistical significance was defined as α = 0.05 beforehand. Analyses were performed in the GraphPad Prism 8 or 10 software.

## Results

### Rat β cells express dual oxidases

**Figure 1.**
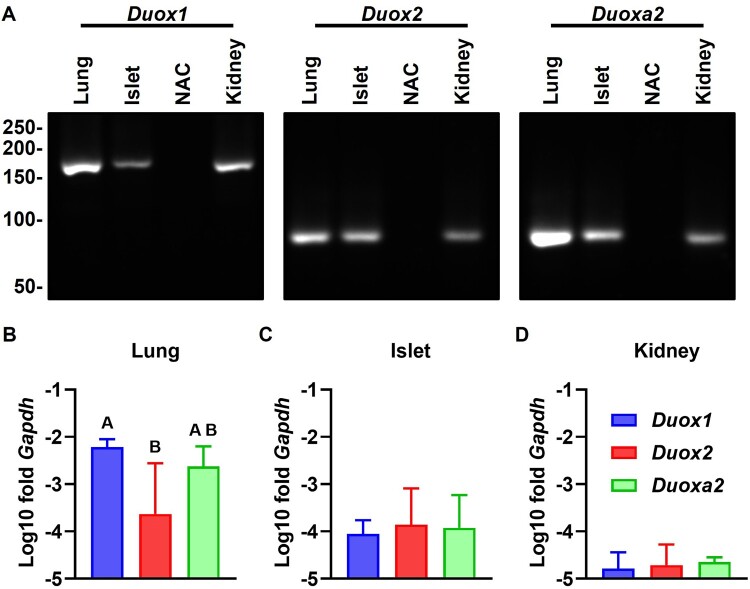
**Pancreatic islets express *Duox(a)* transcripts.** Expression of *Duox1*, *Duox2* and *Duoxa2* in rat islets, lungs and kidneys was measured by RT-qPCR. Dissociation curves are shown in Additional File 7 (Supplementary Figure S1). (A) Representative qPCR reactions fractionated by electrophoresis. Predicted size of amplicons: 166 bp (*Duox1*), 83 bp (*Duox2*) and 87 bp (*Duoxa2*). Molecular weight in bp shown to the left. Gel cropped for clarity. Full-length gels are shown in Additional File 7 (Supplementary Figure S2). (B–D) Expression normalized by *Gapdh*. Blue: *Duox1*, red: *Duox2*, green: *Duoxa2*. Mean ± SD of log_10_-transformed 2^-ΔCT^ values. N = 4. Letters: differences between groups (*p* < 0.05, one-way ANOVA with Tukey’s test).

**Figure 2.**
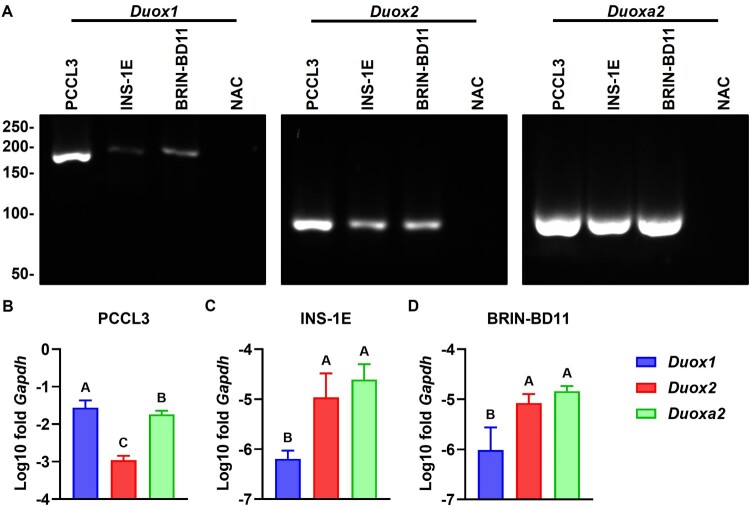
**β cell lines express *Duox(a)* transcripts**. Expression of *Duox1*, *Duox2* and *Duoxa2* in two rat β cell lines (INS-1E and BRIN-BD11) and a rat thyroid cell line (PCCL3) was measured by RT-qPCR. Dissociation curves are shown in Additional File 7 (Supplementary Figure S1). (A) Representative qPCR reactions fractionated by electrophoresis. Predicted size of amplicons: 166 bp (*Duox1*), 83 bp (*Duox2*) and 87 bp (*Duoxa2*). Molecular weight in bp shown to the left. Gel cropped for clarity. Full-length gels are shown in Additional File 7 (Supplementary Figure S2). (B–D) Expression normalized by *Gapdh*. Blue: *Duox1*, red: *Duox2*, green: *Duoxa2*. Mean ± SD of log_10_-transformed 2^-ΔCT^ values. N = 6–8. Letters: differences between groups (*p* < 0.05, one-way ANOVA with Tukey’s test).

Expression of *Duox1*, *Duox2* and *Duoxa2* in rat pancreatic islets (Figure 1) and rat β cell lines (Figure 2) were observed by RT-qPCR. In dissociation curves, all samples displayed peak dF/dT values at similar temperatures (Additional File 7: Supplementary Figure S1). In electrophoresis, the apparent molecular weight of amplified bands was compatible with the predicted size of amplicons: 176 ± 11 bp for *Duox1* (predicted: 166 bp), 85 ± 1 bp for *Duox2* (predicted 83 bp) and 80 ± 4 bp for *Duoxa2* (predicted: 87 bp) (Figures 1 and 2(A)). As expected, no amplification was detected in NTC and NAC reactions. Relative to *Gapdh*, *Duox(a)* expression in islets (−3.95 ± 0.57, Figure 1(C)) was lower than in lungs (−2.83 ± 0.87, Figure 1(B), high-expression control) and higher than in kidneys (−4.72 ± 0.30, Figure 1(D), low-expression control). Expression in INS-1E (−5.26 ± 0.77, Figure 2(C)) and BRIN-BD11 (−5.31 ± 0.59, Figure 2(D)) cells was lower than in the PCCL3 thyroid cell line (−2.09 ± 0.65, Figure 2(B), high-expression control). *Duox(a)* transcript levels are similar in islets (F = 0.1039, *p* = 0.9024, Figure 1(C)); while *Duox1* is lower than *Duox(a)2* in INS-1E (F = 35.46, Figure 2(C)) and BRIN-BD11 (F = 28.29, Figure 2(D)). This contrasts with lungs and PCCL3, where *Duox2* is lower than *Duox1* and *Duoxa2* (F = 4.651, Figure 1(B), and F = 230.5, Figure 2(B)).

**Figure 3.**
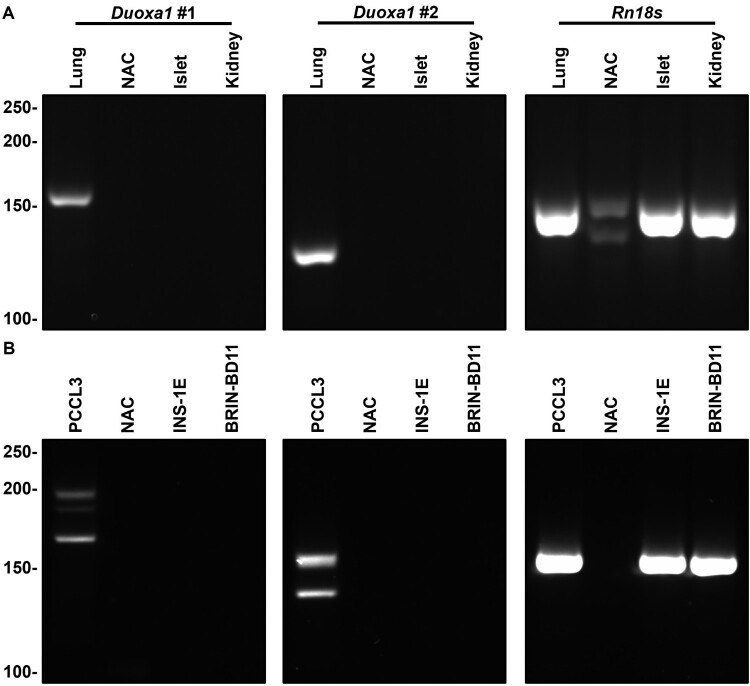
**Rat β cells do not express *Duoxa1* transcripts**. Expression of *Duoxa1* in rat islets and two β cell lines (INS-1E and BRIN-BD11) was analysed by RT-qPCR with two pairs of primers: *Duoxa1#1* (left panels) and *Duoxa1#2* (center panels). The *Rn18s* gene (right panels) was used as a positive amplification control; as well as lungs and the thyroid cell line PCCL3 for tissues and cell lines, respectively. Kidneys were used as negative amplification controls for tissues. Dissociation curves are shown in Additional File 7 (Supplementary Figures S3). Representative qPCR reactions from tissues (A) and cell lines (B) fractionated by electrophoresis. Representative No Amplification Control (NAC) reactions from lungs and PCCL3 are shown. Predicted size of amplicons: 178 bp (*Duoxa1#1*), 144 bp (*Duoxa1#2*) and 167 bp (*Rn18s*). Molecular weight in bp shown to the left. Gels cropped for clarity. The full-length gels are shown in Additional File 7 (Supplementary Figure S4).

Expression of *Duoxa1* was not detected in rat pancreatic islets and INS-1E and BRIN-BD11 by RT-qPCR using two pairs of specific primers (Figure 3). Dissociation curves indicate no amplification of *Duoxa1* in islets, kidneys, INS-1E and BRIN-BD11 samples, as well as in NTC and NAC reactions (Additional File 7: Supplementary Figure S3). This is in opposition to amplification of *Duoxa1* in lungs and PCCL3, confirming primer specificity (Additional File 7: Supplementary Figure S3); and amplification of *Rn18s* in islets, kidneys, INS-1E and BRIN-BD11, confirming cDNA integrity (Additional File 7: Supplementary Figure S3). Electrophoresis confirms the absence of products in representative samples (Figure 3). Bands somewhat matched the predicted size of amplicons: 196, 186 and 166 bp for *Duoxa1*#1 (predicted: 178 bp), 154 and 135 bp for *Duoxa1*#2 (predicted: 144 bp); and 149 ± 1 bp for *Rn18s* (predicted: 167 bp) (Figure 3). Detection of multiple bands with distinct weights in electrophoresis (Figure 3) might represent splicing variants of *Duoxa1*. Alternative splicing of this transcript is documented for the human genome [10,11] and the RefSeq annotation of the *R. norvegicus* genome (NCBI Reference Sequence: NC_086021.1) predicts variants of the transcript (GenBank ID: 311374).

**Figure 4.**
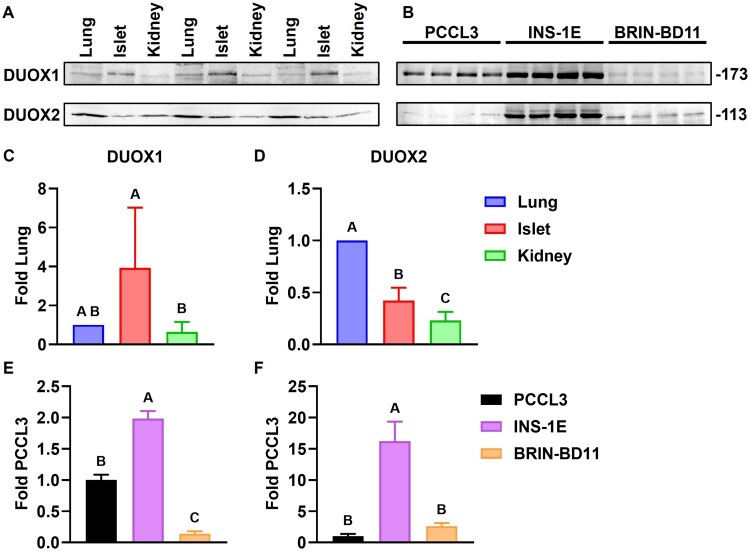
**Rat β cells express DUOX proteins**. Expression of DUOX1 and DUOX2 in rat islets and two rat β cell lines (INS-1E and BRIN-BD11) was analysed by immunoblotting. Lungs and the thyroid cell line PCCL3 were used as positive controls for tissues and cell lines, respectively; kidneys were used as negative controls for tissues. (A–B) Representative blots of three biological replicates from tissues (A) and four biological replicates from cell lines (B). Molecular weight in kDa is shown to the right. Blots cropped for clarity. Full-length blots are shown in Additional File 7 (Supplementary Figures S5–6). (C–F) Expression of DUOX1 (C and E) and DUOX2 (D and F) in tissues (C–D) and cell lines (E–F) normalized by Ponceau S staining and lungs (for tissues) or PCCL3 (for cell lines). Blue: lungs, red: islets, green: kidneys; black: PCCL3, purple: INS-1E, orange: BRIN-BD11. Mean ± SD, N = 4–5. Letters: differences between groups (*p* < 0.05, one-way ANOVA with Tukey’s test).

**Figure 5.**
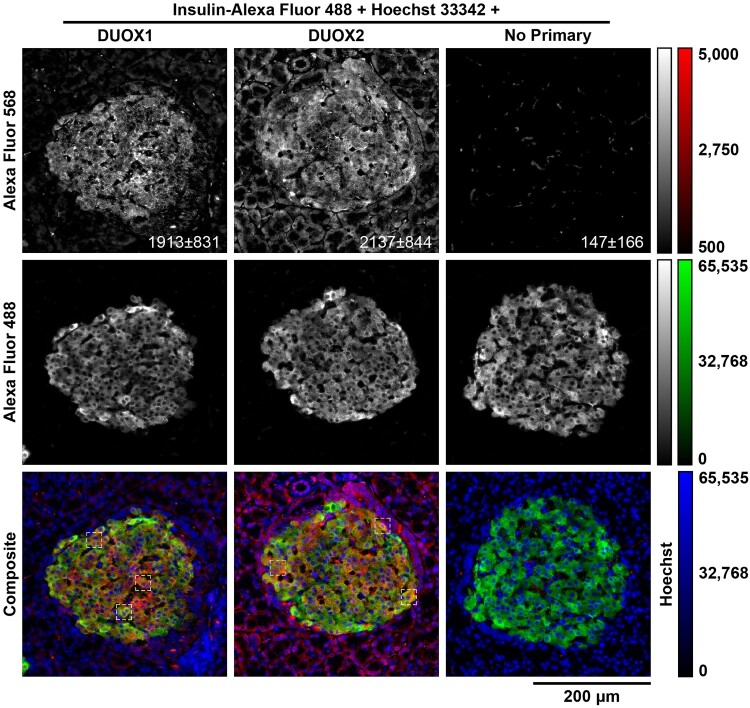
**Expression of DUOX proteins in islets**. Widefield fluorescent microscopy of rat pancreata sections (6 μm) indirectly stained with anti-DUOX1 (1:100, left column) or anti-DUOX2 (1:100, middle column) and Alexa Fluor 568 (1:500) and directly stained with anti-insulin-Alexa Fluor 488 (1:100) and Hoechst 33342. Controls were incubated without DUOX antibodies (No Primary, right column). Images were captured with a LD Plan-Neofluar 20×/0.4 Korr Ph 2 objective with constant exposure of 3.0 seg. Representative images displayed as single-channel grayscales for Alexa Fluor 568 (top row) and Alexa Fluor 488 (middle row) or as three-channel false-color composites (bottom row). Red: Alexa Fluor 568, green: Alexa Fluor 488, blue: Hoechst 33342. Red channels displayed with identical linear contrast-stretching for all images (from 500 to 5.000 intensity) to facilitate visualization. Grayscale and false-color calibration keys shown to the right. Scale bar: 200 μm. Mean ± SD pixel intensity of red channels within insulin-positive areas shown at the bottom right corner of images. Dashed boxes: insets in Figure 6.

**Figure 6.**
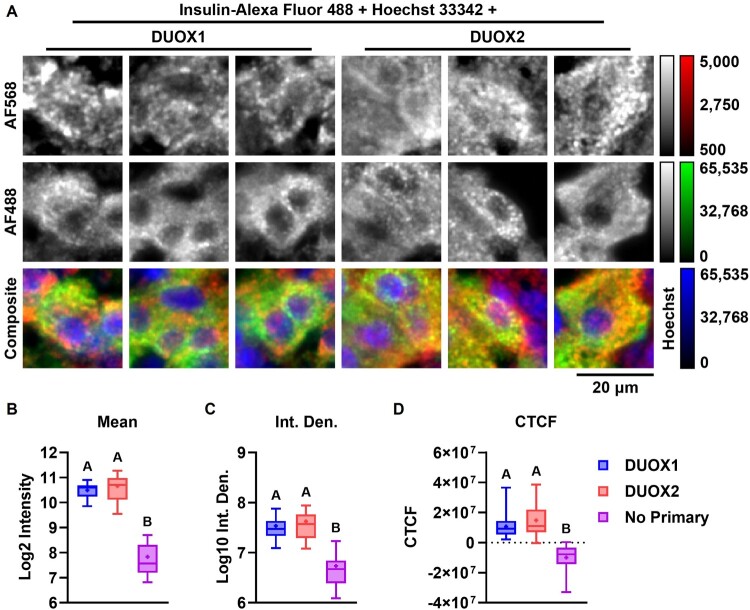
**DUOX staining in insulin-positive islet cells**. (A) Insets shown in Figure 5 of pancreata sections stained for DUOX1/2 with Alexa fluor 568, insulin with Alexa Fluor 488 and nuclei with Hoechst 33342. Images displayed as single-channel grayscales for Alexa Fluor 568 (top row) and Alexa Fluor 488 (middle row) or as three-channel false-color composites (bottom row). Red: Alexa Fluor 568, green: Alexa Fluor 488, blue: Hoechst 33342. Red channels displayed with identical linear contrast-stretching for all images (from 500 to 5.000 intensity) to facilitate visualization. Grayscale and false-color calibration keys shown to the right. Scale bar: 20 μm. (B–D) Quantification of DUOX staining within insulin-positive areas. (B) Log_2_ mean pixel intensity. (C) Log_10_ integrated density (Int. Den.). (D) Corrected total cell fluorescence (CTCF). Min-Max box plots, mean indicated by + . N = 18. Blue: DUOX1, red: DUOX2, purple: No Primary control. Letters: differences between groups (*p* < 0.05, one-way ANOVA with Tukey’s test).

**Figure 7.**
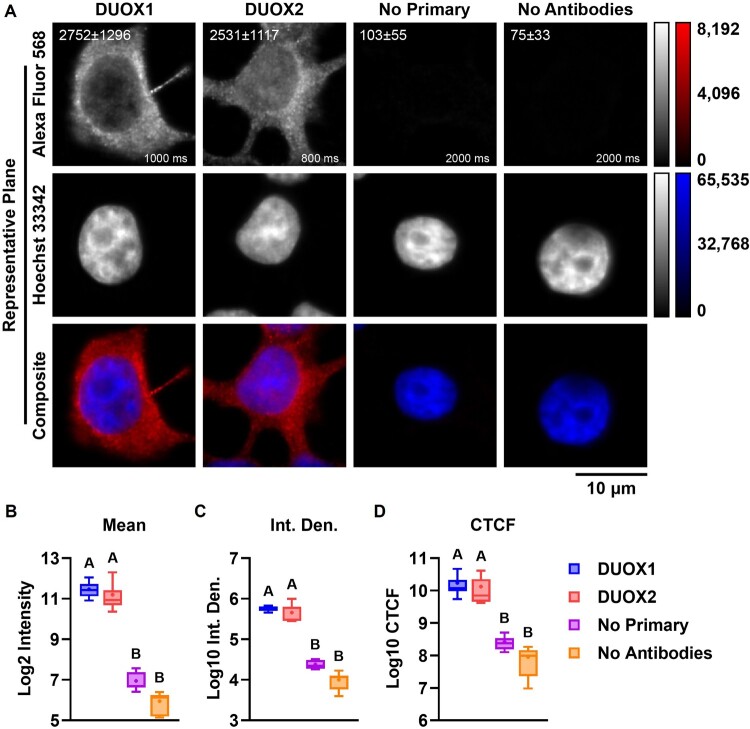
**Expression of DUOX proteins in INS-1E**. (A) Widefield fluorescent microscopy of INS-1E indirectly stained with anti-DUOX1 (1:100, first column) or anti-DUOX2 (1:100, second column) and Alexa Fluor 568 (1:500) and Hoechst 33342. Controls were incubated without primary antibodies (No Primary, third column) or without primary and without secondary antibodies (No Antibodies, fourth column). Images were captured in Z-stacks (0.24 μm intervals) with a Plan-Apochromat 100×/1.4 Oil objective. Single representative planes displayed as single-channel grayscales for Alexa Fluor 568 (top row) and Hoechst 33342 (middle row) or two-channel false-color composites (bottom row). Red: Alexa Fluor 568, blue: Hoechst 33342. Red channels displayed with identical linear contrast-stretching for all images (from 0 to 8.192 intensity) to facilitate visualization. Grayscale and false-color calibration keys shown to the right. Scale bar: 10 μm. Mean ± SD pixel intensity (top left) and exposure in ms (bottom right) for red channels shown for each image. (B–D) Quantification of DUOX staining in within cells. (B) Log_2_ mean pixel intensity. (C) Log_10_ integrated density (Int. Den.). (D) Corrected total cell fluorescence (CTCF). Min-Max box plots, mean indicated by + . N = 9. Blue: DUOX1, red: DUOX2, purple: No Primary control, orange: No Antibodies control. Letters: differences between groups (*p* < 0.05, one-way ANOVA with Tukey’s test).

Protein expression of DUOX1 and DUOX2 in islets, INS-1E and BRIN-BD11 is demonstrated quantitatively by immunoblotting (Figure 4(A and B)) and qualitatively by immunohistochemistry (Figures 5 and 6) and by immunocytochemistry (Figure 7). DUOX1 expression in islets (3.93 ± 3.04) is similar to that in lungs and significantly higher than in kidneys (0.64 ± 0.51) (Figure 4(C)). DUOX2 expression in islets (0.42 ± 0.12) is lower than in lungs and higher than in kidneys (0.23 ± 0.08) (Figure 4(D)). Relative to PCCL3, DUOX1 expression is significantly higher in INS-1E (1.98 ± 0.12) and significantly lower in BRIN-BD11 (0.14 ± 0.04) (Figure 4(E)). DUOX2 expression is also significantly higher in INS-1E (16.23 ± 3.11). DUOX2 expression in BRIN-BD11 (2.6 ± 0.5) is similar to that in PCCL3 (Figure 4(F)). Staining with anti-DUOX1 and anti-DUOX2 in whole islets (Figures 5 and 6), β cells *in situ* (Figure 6(A)) and INS-1E (Figure 7(A)) is shown. Mean px intensity (Figures 6 and 7(B)), integrated density (Figures 6 and 7(C)) and CTCF (Figures 6 and 7(D)) in insulin-positive areas (Figure 6) and INS-1E (Figure 7) is significantly brighter for anti-DUOX1 and anti-DUOX2, relative to antibody controls and autofluorescence controls. Of note, anti-DUOXA1 and anti-DUOXA2 antibodies were not commercially available at the time experiments were performed.

### Localization of NOX isoforms

**Figure 8.**
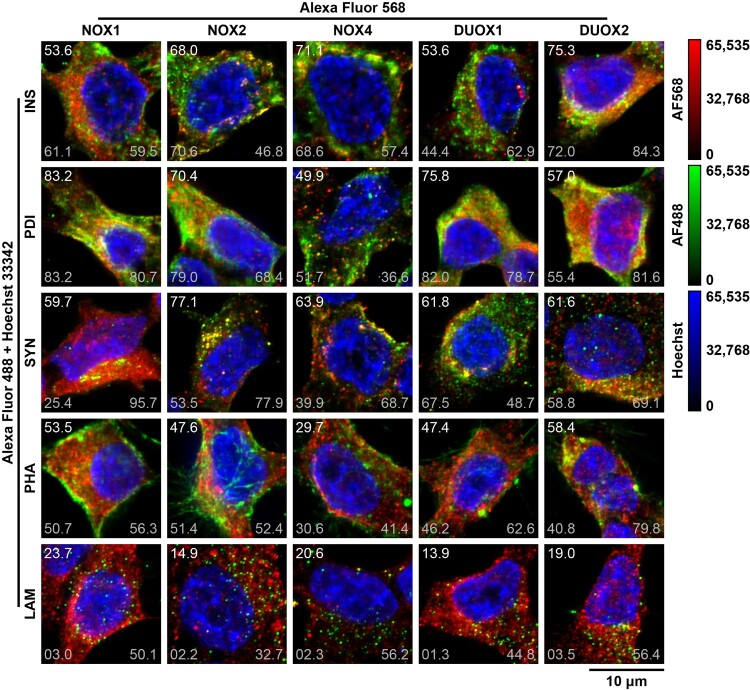
**Localization of NOX isoforms**. Widefield fluorescent microscopy of INS-1E. Cells were stained for NOX isoforms (1:100–200) with Alexa Fluor 568 (1:500); and co-stained for insulin (INS), PDI, syntaxin 1 (SYN), F-actin (PHA) and LAMP1 (LAM) (1:100–400) with Alexa Fluor 488 (1:500) and DNA with Hoechst 33342 for co-labeling of insulin vesicles, the ER, plasma membrane, actin cytoskeleton, lysosomes and nuclei, respectively. Images were captured as Z-stacks (0.24 μm intervals) with a Plan-Apochromat 100×/1.4 Oil objective and deconvoluted and processed digitally. Representative stacks displayed as maximum-intensity projections of 10 planes in three-channel false-color composites. Red: Alexa Fluor 568, green: Alexa Fluor 488, blue: Hoechst 33342. For red × green comparisons, Pearson’s correlation coefficient (top right) and Manders’ colocalization coefficients M1 (bottom right) and M2 (bottom left) are shown × 100 for each stack. False-color calibration keys shown to the right. Scale bar: 10 μm.

**Figure 9.**
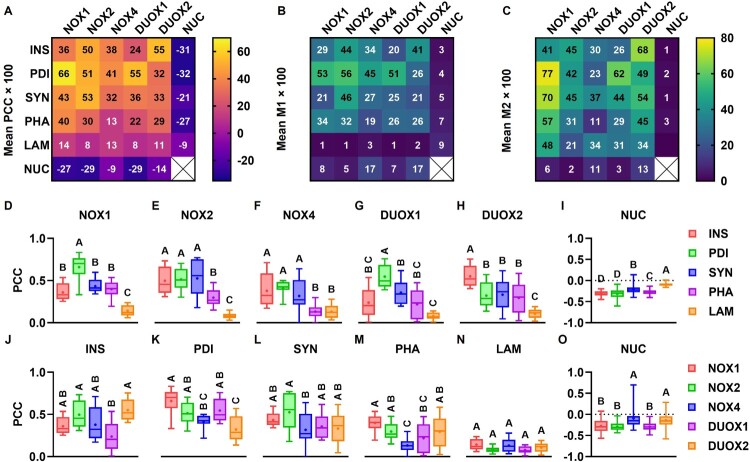
**Correlation and colocalization analyses**. INS-1E were stained for NOX isoforms with Alexa Fluor 568; and co-stained for insulin (INS), PDI, syntaxin 1 (SYN), F-actin (PHA) and LAMP1 (LAM) with Alexa Fluor 488 and DNA with Hoechst 33342 (NUC) for labeling of insulin vesicles, the ER, plasma membrane, actin cytoskeleton, lysosomes and nuclei, respectively, for immunocytochemistry (Figure 8). (A–C) Association between fluorophores measured by Pearson’s correlation coefficient (PCC, A) and Manders’ colocalization coefficients M1 (B) and M2 (C) for all pairs, each labeled with its respective mean × 100. Color scales shown to the right of heat maps. (D–H) PCC of indicated isoforms (D–H) and nuclei (I), grouped according to intracellular domain. Red: insulin vesicles, green: ER, blue: plasma membrane, purple: actin cytoskeleton, orange: lysosomes. (J–O) PCC of indicated intracellular domains, grouped according to isoform. Red: NOX1, green: NOX2, blue: NOX4, purple: DUOX1, orange: DUOX2. Min-Max box plots, mean indicated by + . Letters: differences between groups (*p* < 0.05, one-way ANOVA with Tukey’s test). (D–H and J–N) N = 8–12. (I and O) N = 44–72.

NOX isoforms were stained with Alexa Fluor 568 in INS-1E (Figure 8). Insulin, PDI, syntaxin 1, F-actin and LAMP1 were co-stained with Alexa Fluor 488 and DNA was co-stained with Hoechst 33342 for simultaneous labeling of insulin vesicles, ER, plasma membrane, actin cytoskeleton, lysosomes and nuclei, respectively. Association between fluorophores was measured by PCC (correlation), M1 and M2 (colocalizations) (Figure 9(A–C)). Associations between Alexa Fluor 488 and Hoechst 33342 were significantly different from zero (Additional File 5: Association). The respective correlations were negative and (very) weak (PCC ≤ −0.09, Figure 9(A)) and colocalizations were low: M1 ≤ 8.6% (Figure 9(B)); M2 ≤ 2.5% (Figure 9(C)). This indicates the absence of insulin, PDI, syntaxin 1, F-actin and LAMP1 in nuclei.

For all associations involving isoforms, only the M1 for NOX4 × LAMP1 was not significantly different from zero (Additional File 5: Association). Correlation of NOX1, NOX2 and DUOX1 with DNA was negative and weak (PCC ≤ −0.27) and colocalization was low (M1 ≤ 7.9% and M2 ≤ 6.1%, Figure 9(A)). This supports the absence of these isoforms in nuclei, as indicated by visual analysis of images (Figure 8). However, NOX4 and DUOX2 occasionally stained the nucleus in some specimens (Figure 8), causing a greater dispersion in the correlation data (F = 24.66, Figure 9(O)). Relative to other isoforms, their correlation with DNA was significantly higher (Figure 9(O)), as for their colocalization within (Figure 9(B)) and with (Figure 9(C)) DNA (statistical analysis not shown). Thus, our data do not support the complete nuclear absence of NOX4 and DUOX2. Although NOX4 and DUOX2 are not nuclear residents *per se*, the nucleus might be permissible to the presence of these isoforms.

All other correlations were positive. All isoforms correlated weakly or very weakly with F-actin and very weakly with LAMP1; with correspondingly low localization within F-actin (M1 ≤ 34.1%) and LAMP1 (M1 ≤ 3.3%) (Figure 9(A)). NOX1 correlated strongly with PDI (0.66 ± 0.16) and weakly with insulin (0.36 ± 0.11). NOX4 and DUOX1 correlated weakly with insulin (0.38 ± 0.20 and 0.24 ± 0.18, respectively) and with syntaxin 1 (0.32 ± 0.21 and 0.36 ± 0.14). DUOX2 correlated weakly with PDI (0.32 ± 0.15) and with syntaxin 1 (0.33 ± 0.17). All the remaining correlations were moderate. No pair had very strong (|r| ≥ 0.80) correlation, probably due to limited resolution, noise in the data or off-targeting by NOX antibodies [45].

Associations were compared via ANOVA with Tukey’s test amongst intracellular domains within isoforms (Figure 9(D–H)) and amongst isoforms within intracellular domains (Figure 9(J–O)). Relative to other domains, NOX1 (F = 36.98) and DUOX1 (F = 19.32) preferentially correlate with the ER and DUOX2 (F = 14.71) preferentially correlates with insulin vesicles. NOX2 (F = 22.15) and NOX4 (F = 8.77) correlate with insulin vesicles, the ER and syntaxin 1 similarly. Relative to other isoforms, insulin correlates the most with DUOX2 and the least with DUOX1 (F = 5.8); PDI correlates the most with NOX1 and the least with DUOX2 (F = 10.31); syntaxin 1 correlates the most with NOX2 and the least with NOX4 (F = 3.02); and F-actin correlates the most with NOX1 and the least with NOX4 (F = 7.11).

For M1, NOX1 localized mostly within PDI (53 ± 23%); NOX2 within PDI (56 ± 13%), syntaxin 1 (46 ± 22%) and insulin (44 ± 17%); NOX4 and DUOX2 within PDI (45 ± 6% and 26 ± 12%respectively) and insulin (34 ± 21% and 41 ± 15%); and DUOX1 within PDI (51 ± 17%) (Figure 9(B)). For M2, insulin colocalized mostly with DUOX2 (68 ± 16%) and NOX2 (45 ± 20%); PDI with NOX1 (77 ± 8%) and DUOX1 (62 ± 11%); syntaxin 1 with NOX1 (70 ± 15%) and DUOX2 (54 ± 27%); F-actin with NOX1 (57 ± 10%) and DUOX2 (45 ± 18%); LAMP1 with NOX1 (48 ± 14%), NOX4 (34 ± 15%) and DUOX2 (34 ± 13%); and DNA with DUOX2 (13 ± 15%) and NOX4 (11 ± 19%) (Figure 9(C)).

**Figure 10.**
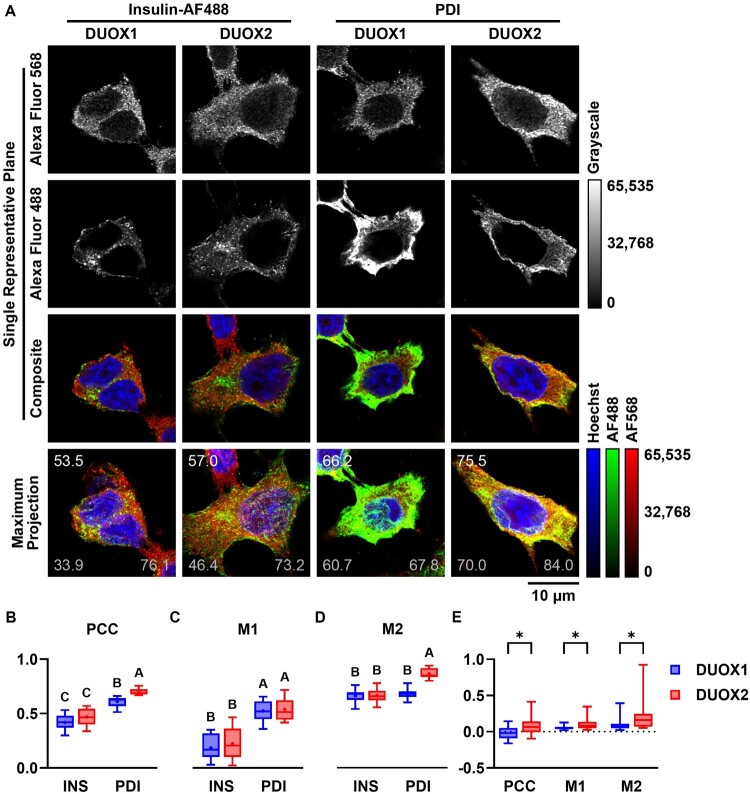
**Localization of DUOX isoforms**. (A) CLSM of INS-1E. Cells were indirectly stained with anti-DUOX1 (1:100, first and third columns) or anti-DUOX2 (1:100, second and fourth columns) and Alexa Fluor 568 (1:500); directly stained with insulin-Alexa Fluor 488 (1:100, first and second columns) or indirectly stained with PDI (1:100, third and fourth columns) and Alexa Fluor 488 (1:500) and Hoechst 33342 for co-labeling of insulin vesicles, ER and nuclei, respectively. Images were captured as Z-stacks (0.28 μm intervals) with a HC PL APO CS2 63×/1.4 Oil objective in 3× optical zoom, deconvoluted and processed digitally. Single representative planes of representative stacks displayed as grayscales for Alexa Fluor 568 (first row) and Alexa Fluor 488 (second row) or as three-channel false-color composites (third row). Maximum-intensity projections of 11 planes of respective stacks displayed as three-channel false-color composites (fourth row). Red: Alexa Fluor 568, green: Alexa Fluor 488, blue: Hoechst 33342. Grayscale and false-color calibration keys shown to the right. Scale bar: 10 μm. For red × green comparisons, Pearson’s correlation coefficient (PCC, top right) and Manders Colocalization Coefficients M1 (bottom right) and M2 (bottom left) are shown × 100 for each stack. (B–E) PCC (B and E), M1 (C and E) and M2 (D and E) for red × green (B–D) and red × blue (E) comparisons. Min-Max box plots, mean indicated by + . Blue: DUOX1, red: DUOX2. (B–D) Letters: differences between groups (*p* < 0.05, two-way ANOVA with Tukey’s test). N = 10. (E) *p < 0.05 (Holm-Šídák multiple unpaired t tests). N = 20.

Since DUOX1 and DUOX2 associated mostly with the ER and insulin, respectively, we sought to investigate such associations further in CSLM (Figure 10). INS-1E were stained for DUOX1 or DUOX2 and co-stained for insulin or PDI (Figure 10(A)). PCC, M1 and M2 were measured as described above. Except for the M1 of DUOX1 × Hoechst 33342, all associations were significantly different from zero (Additional File 5: Association). Correlation of both isoforms is moderate for insulin and strong for PDI (Figure 10(B)). Both isoforms correlated very weakly with nuclei, despite DUOX2 having significantly higher PCC (0.08 ± 0.12), M1 (11 ± 8%) and M2 (22 ± 23%) relative to DUOX1 (PCC = −0.02 ± 0.09, M1 = 5 ± 3%, M2 = 10 ± 8%) (Figure 10(E)). Associations were further compared across isoforms and across domains (two-way ANOVA with Tukey’s test). For PCC, both isoforms correlated similarly with insulin (0.42 ± 0.07 for DUOX1 and 0.47 ± 0.08 for DUOX2), while DUOX2 correlated significantly more with PDI (0.70 ± 0.03) relative to DUOX1 (0.61 ± 0.05) (F = 13.59 for isoform, F = 122.8 for domain, Figure 10(B)). For M1, both isoforms localized similarly within insulin (18 ± 12% for DUOX1 and 22 ± 14 for DUOX2) and PDI (52 ± 10% for DUOX1 and 54 ± 11% for DUOX2), despite a significantly higher localization within the latter (F = 0.5 for isoform, F = 77.85 for domain, Figure 10(C)). For M2, insulin colocalized similarly with both isoforms (66 ± 7% for DUOX1 and 66 ± 6% for DUOX2) and PDI colocalized significantly more with DUOX2 (86 ± 5%) relative to DUOX1 (68 ± 5%) (F = 26.78 for isoform, F = 38.14 for domain, Figure 10(D)).

### Regulation of NOX expression

**Figure 11.**
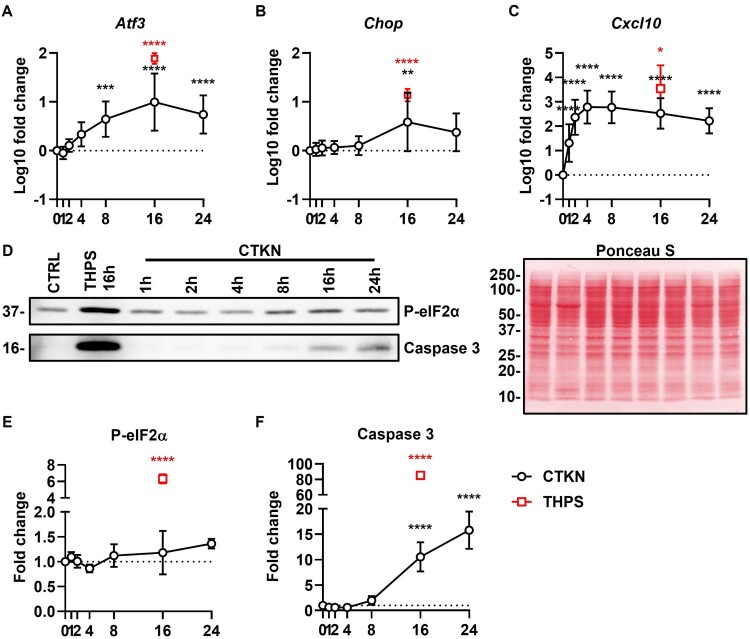
**Cellular response to insults**. INS-1E were treated with cytokines (CTKN, black) or thapsigargin (THPS, red) for the indicated time periods or left untreated (0 h). (A–C) *Atf3*, *Chop* and *Cxcl10* expression measured by RT-qPCR, normalized by *Rn18s* and untreated cells. Mean ± SD of log_10_-transformed 2^-ΔΔCT^ values, N = 5–8. (D) Representative blots for P-eIF2α and caspase 3 (cropped from different experiments). One representative Ponceau S staining is shown in false-color. Full-length blots are shown in Additional File 7 (Supplementary Figure S7). Molecular weight in kDa shown to the left. (E–F) Expression of P-eIF2α and caspase 3 measured by immunoblotting, normalized by Ponceau S staining and untreated cells. Mean ± SD, N = 3. Dotted lines indicate untreated cells. *p < 0.05, **p < 0.01, ***p < 0.001, ****p < 0.0001 vs. untreated cells. Mixed-effects model (A–C) or RM one-way ANOVA (E–F) with Dunnett’s test.

Exposure to thapsigargin and cytokines increased mRNA expression of *Atf3*, *Chop* and phosphorylation of eIF2α (markers of ER stress); mRNA expression of *Cxcl10* (marker of chemokine release); and activation of caspase 3 (marker of apoptosis) (Figure 11). This indicates a typical β cell response to the respective insults [56–58,67].

**Figure 12.**
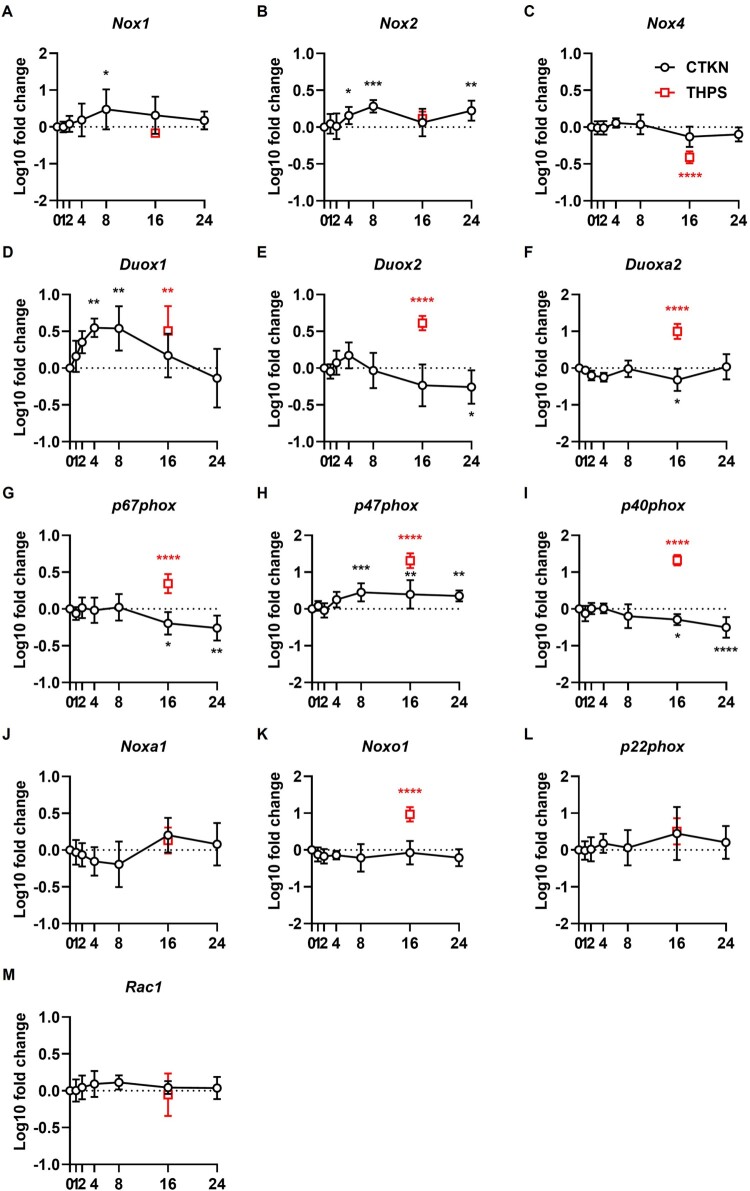
**Regulation of *Nox* transcripts**. INS-1E were treated with cytokines (CTKN, black) or thapsigargin (THPS, red) for the indicated time periods or left untreated (0 h). Expression of indicated *Nox* genes measured by RT-qPCR, normalized by *Rn18s* and untreated cells. Mean ± SD of log_10_-transformed 2^-ΔΔCT^ values, N = 4–5. Dotted lines indicate untreated cells. *p < 0.05, **p < 0.01, ***p < 0.001, ****p < 0.0001 vs. untreated cells. Mixed-effects models (A–B, G–H, M) or RM one-way ANOVA (C–F, I–L) with Dunnett’s test.

**Figure 13.**
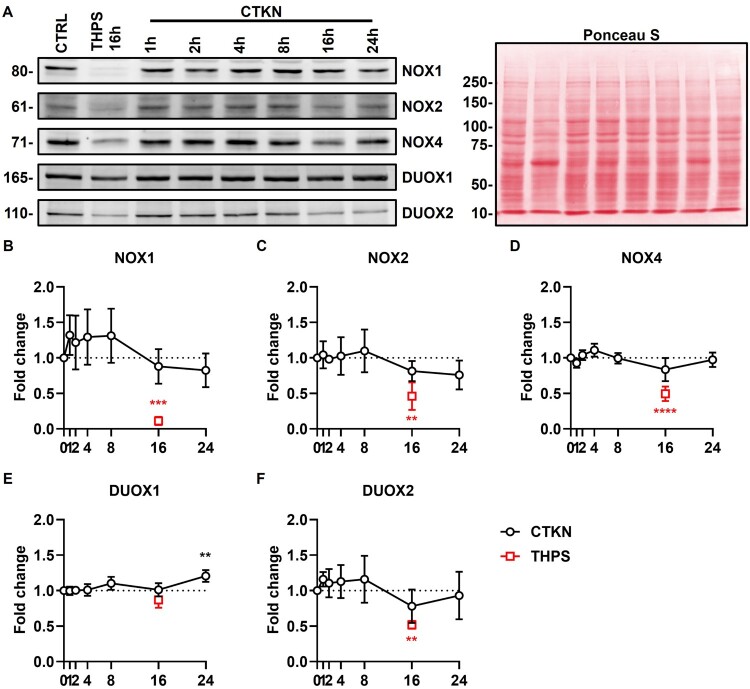
**Regulation of NOX proteins**. INS-1E were treated with cytokines (CTKN, black) or thapsigargin (THPS, red) for the indicated time periods or left untreated (0 h). (A) Representative blots for each isoform (cropped from different experiments). One representative Ponceau S staining is shown in false color. Full-length blots are shown in Additional File 7 (Supplementary Figure S7). Molecular weight in kDa indicated to the left. (B–F) Expression of indicated NOX proteins normalized by Ponceau S staining and untreated cells. Mean ± SD, N = 3. Dotted lines indicate untreated cells. **p < 0.01, ***p < 0.001, ****p < 0.0001 vs. untreated cells (RM one-way ANOVA with Dunnett’s test).

Thapsigargin significantly upregulated mRNA expression of the regulatory factors *p67phox*, *p47phox*, *p40phox* and *Noxo1* and the isoforms *Duox1* and *Duox2*; alongside its scaffolding partner *Duoxa2* (Figure 12). Thapsigargin significantly downregulated NOX4 (mRNA and protein) (Figures 12 and 13(C)) and protein expression of NOX1, NOX2 and DUOX2 (Figure 13).

Cytokines significantly upregulated mRNA expression of the regulatory factor *p47phox* (8–24 h) and the isoforms *Nox1* (8 h), *Nox2* (4–24 h) and *Duox1* (4–8 h) (Figure 12) and protein expression of DUOX1 (24 h) (Figure 13(E)). Cytokine upregulation of NOX1 (1–8 h) protein expression was small (≈ 30%) and non-statistically significant (Figure 13(B)). Cytokines significantly downregulated mRNA expression of the regulatory factors *p67phox* and *p40phox* (16–24 h), the isoform *Duox2* (24 h) and its scaffolding partner *Duoxa2* (16 h) (Figure 12). Cytokine downregulation of NOX2 (16–24 h) and DUOX2 (16 h) protein expression was small (≈ 20%) and non-statistically significant (Figure 13).

Log_10_-transformed 2^-ΔCT^ values (relative to *Rn18s*) in untreated INS-1E were used to compare the mRNA expression of isoforms and subunits (Additional File 6: Treatment). Among isoforms, *Nox4* has the highest expression (−4.92 ± 0.11); followed by *Duox2* (−5.75 ± 0.25) and *Nox2* (−6.04 ± 0.24); and followed by very low expression of *Duox1* (−7.22 ± 0.24) and *Nox1* (−7.30 ± 0.25). Among subunits, *Rac1* has the highest expression (−2.55 ± 0.25); followed by *Noxa1* (−5.65 ± 0.18), *Duoxa2* (−5.74 ± 0.2), *p47phox* (−6.45 ± 0.28), *Noxo1* (−6.47 ± 0.15), *p67phox* (−6.58 ± 0.2), *p40phox* (−6.99 ± 0.13) and *p22phox* (−7.21 ± 0.15).

Of note, we did not investigate whether thapsigargin and cytokines induce *Duoxa1* mRNA expression, which is demonstrably undetectable in untreated β cells (Figure 3). Also, immunoblotting data might be partially biased due to antibody off-targeting. No commercially available anti-NOX antibodies are free from this problem [18,45].

## Discussion

We characterized NOX expression in β cells, focusing on their subcellular localization and time-dependent regulation by cytokines. Expression of *Duox1* and *Duox(a)2* transcripts (Figures 1 and 2); the absence of *Duoxa1* transcripts (Figure 3); and expression of DUOX1 and DUOX2 proteins (Figures 5–7) in rat pancreatic islets and two rat β cell lines (INS-1E and BRIN-BD11) is shown for the first time. Interestingly, *Duox1* and *Duoxa1* share a common bidirectional promoter in Mammalia [10,68] and are typically co-expressed in other cells, such as thyrocytes and epithelial cells [4,7]. Hence, expression of *Duox1*, but not of *Duoxa1*, in β cells is unexpected and may indicate that *Duoxa1* is a ‘*disallowed gene’* – a term referring to genes that are transcriptionally repressed in β cells in order to maintain their identity and function [69–71].

**Figure 14.**
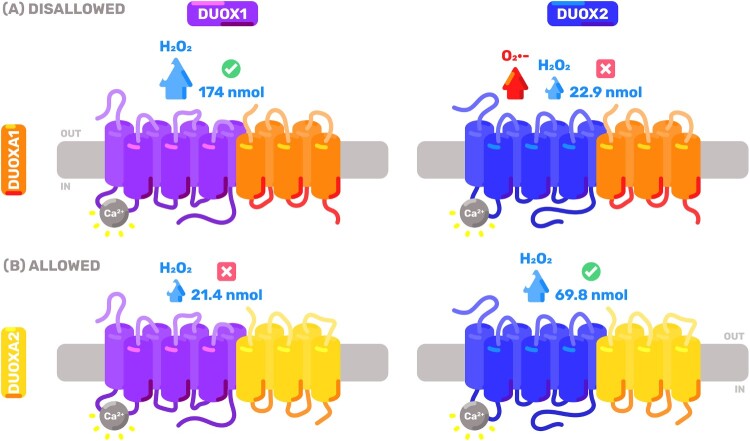
**Speculative model of dual oxidase configurations in β cells**. The four possible configurations of dual oxidases are represented. DUOX1 (purple) and DUOX2 (blue) associate with either DUOXA1 (orange) or DUOXA2 (yellow) in *matched* (green checkbox) or *mismatched* (red checkbox) heterodimers. Mismatching impairs enzyme maturation, trafficking and kinetics. Stimulated H_2_O_2_ production represented in cyan and $O_{2}^{∙-}$ leakage in red. Numbers indicate kinetics of stimulated H_2_O_2_ production in nmol/h/10^6^ cells (see main text for reference). Given the absence of *Duoxa1* in β cells, the ‘*Disallowed*’ (A) heterodimers (top row) are not present and only the ‘*Allowed*’ (B) heterodimers (bottom row) are speculated to be present in this cell type.

DUOX:DUOXA heterodimers can exist in *matched* (DUOX1:DUOXA1 and DUOX2:DUOXA2) and *mismatched* (DUOX1:DUOXA2 and DUOX2:DUOXA1) configurations (Figure 14). Mismatching impairs organelle trafficking, leading to increased retention in the ER and Golgi and decreased trafficking to other sites (such as the plasma membrane) [11,12]. It also impairs their enzymatic activity: stimulated H_2_O_2_ production is decreased for both isoforms and $O_{2}^{∙-}$ leakage becomes permissible for DUOX2 (Figure 14) [11,12,72].

Given the absence of *Duoxa1* in β cells, DUOX1 may only exist in its mismatched configuration and DUOX2 may only exist in its matched configuration in this cell type. Putatively, this scenario might significantly alter the kinetic and functional behavior of DUOX in β cells (Figure 14). First, it prohibits formation of the kinetically-faster configuration (matched DUOX1). Second, it also prohibits formation of the only $O_{2}^{∙-}$-leaking configuration (mismatched DUOX2). Third, it renders DUOX2 three times kinetically faster relative to DUOX1. Fourth, it might impair DUOX1 maturation and trafficking. This raises the hypothesis that the absence of *Duoxa1* in β cells serves as a mechanism for kinetic and spatial control of DUOX activity. Future studies should investigate (i) whether *Duoxa1* is indeed a disallowed gene in β cells; (ii) the mechanisms underlying its transcriptional repression; and (iii) how *Duoxa1* expression – or its absence – impacts DUOX kinetics and β cell biology.

DUOX1 and DUOX2 localize primarily at the apical membrane in thyrocytes and airway epithelial cells – typical models of DUOX-expressing cells [4,7]. In contrast, we showed that DUOX1 and DUOX2 in β cells localize preferentially at the ER and insulin vesicles and less so at the plasma membrane (Figures 8–10), indicating the retention of DUOX1 in the ER due to the absence of *Duoxa1* and suggesting that DUOX2 might be the main NOX isoform in insulin vesicles. Their lesser localization at the plasma membrane might indicate the (i) suboptimal migration of mismatched DUOX1 towards this domain in β cells, consistent with studies in other cell types [11]; and (ii) plasma membrane insertion of DUOX2 during the exocytosis of insulin. Importantly, GSIS depends upon the activity of voltage-gated (VGCCs) and calcium-gated (CGCCs) Ca^2+^ channels primarily located at the plasma membrane and the ER [73,74]. Additionally, VGCCs associate with SNARE proteins in β cells to form *exocytosis signalosomes* at the plasma membrane [74]. Thus, the localization of DUOX1 and DUOX2 at the ER, insulin vesicles and, to a lesser extent, plasma membrane (Figure 9) suggests their proximity to Ca^2+^ channels and signalosomes in β cells.

DUOXs might be important H_2_O_2_ sources in β cells and regulators of GSIS, as suggested by their intracellular localization in INS-1E (Figure 9) and biological properties (activation by Ca^2+^ and regulation by NADPH and Ca^2+^ levels) [7,50,51]. Glucose stimuli probably activate DUOX1 and DUOX2 via ER Ca^2+^ release and plasma membrane Ca^2+^ influx, generating transient and localized H_2_O_2_ bursts. Supporting this, ROS production in β cells is stimulated by glucose and depolarization and both phenomena are sensitive to inhibition by DPI [24–26,28]. Conversely, DUOX activity might regulate Ca^2+^ dynamics in β cells. Namely, Ca^2+^ release from the ER and Ca^2+^ influx through the plasma membrane via redox control of CGCCs, VGCCs and other redox sensitive channels. In other excitable cell types, H_2_O_2_ increases stimulated activity of CGCCS and VGCCs [75] and in β cells, Ca^2+^ signaling is subject to redox control. Exogenous H_2_O_2_ increases cytoplasmic [Ca^2+^], ryanodine receptor activity and insulin secretion [25,28,76]. Antioxidants (such as N-acetyl cysteine, catalase and DPI), decrease glucose-stimulated ROS production, cytoplasmic [Ca^2+^] and insulin secretion [25,32,33]. Thus, the possible interplay between DUOX activity and Ca^2+^ signaling in amplifying insulin secretion is a compelling hypothesis warranting future investigation.

We also show the intracellular localization of NOX1, NOX2 and NOX4 in INS-1E (Figures 8 and 9). Our immunocytochemistry findings challenge the common assumption of NOX isoforms as *exclusive* plasma membrane proteins. Similar to DUOX1, NOX1 is primarily expressed in the ER. In contrast, NOX2 and NOX4 are evenly distributed in insulin vesicles, ER and plasma membrane. This is consistent with their widespread cytoplasmic distribution observed in human islets and the human β cell line EndoC-βH1 [41]. Also, NOX2 colocalization with insulin in INS-1E corroborates previous observations in dispersed human β cells [18]. However, we did not observe NOX2 colocalization with LAMP1 in INS-1E, as reported in dispersed human β cells [18]. We also did not observe NOX4 nuclear staining in INS-1E, as reported in human islets and EndoC-βH1 [41]. In fact, our results indicate the nuclear absence of all isoforms. However, the correlation of NOX4 and DUOX2 with nuclei is significantly less negative relative to other isoforms (Figure 9(O)).

The presence of NOX2, NOX4 and DUOX2 in insulin vesicles and the plasma membrane of INS-1E suggests their proximity to SNAREs in β cells. Local ROS production of vesicle-resident isoforms might impact the assembly and disassembly of SNAREs and consequently regulate vesicle dynamics (docking, priming, fusion) [77]. At neuromuscular junctions, H_2_O_2_ inhibits neurotransmitter release from presynaptic membranes [78,79]. Oxidation of SNAP25 inhibits assembly of SNAREs *in vitro* [79] and induces their partial disassembly *in silico* [80]. Also, *Nox2* and *Nox4* KO increase GSIS without affecting intracellular [Ca^2+^] [18,38,39]. Thus, NOX isoforms might negatively regulate insulin secretion via redox regulation of SNARE dynamics. This inhibitory redox control of SNAREs may function in parallel to the stimulatory redox control of Ca^2+^ dynamics discussed earlier. The dual effects might function in an isoform-specific, bidirectional and time-dependent fashion to fine-tune β cell function: rapid and transient enhancing of Ca^2+^ signaling and delayed sustained dampening of exocytosis via SNARE oxidation.

The expression of NOX1, NOX2 and DUOX1 in the ER of β cells corroborates previous findings from our group of their involvement in cytokine-induced ER stress. KO of *Nox1* prevented cytokine-induced ER stress (Vilas-Boas et al., unpublished data), while *Nox2* KO did not [38]. Instead, early cytokine-induced ER stress precedes and is required for subsequent cytokine-induced activation of NOX2 [38]. In other cells, NOX activity is both an initiator and consequence of ER stress [22]. Mechanistically, NOX activity might induce ER stress via (i) depletion of ER Ca^2+^ stores (via redox regulation of Ca^2+^ channels); (ii) impairment of oxidative folding (via consumption of luminal redox relays and consequent inhibition of foldases and oxidoreductases); and (iii) non-specific oxidative damage of client proteins [22,75,81]. This would result in chaperone exhaustion, impaired ER function, activation of the Unfolded Protein Response (UPR) and apoptosis. Furthermore, the interplay between ROS production and Ca^2+^ release mediate the crosstalk between the UPR and mitochondria during apoptosis [75,82]. Thus, NOXs might regulate the ER-mitochondria apoptotic signaling axis in β cells during inflammation. Consistent with this, both NOX1 and NOX2 are implicated in cytokine-induced β cell death [23,38,83]. The involvement of DUOX1 and DUOX2 in cytokine-induced ER stress and apoptosis in β cells also merits future investigation.

Finally, we examined the time-dependent cytokine-induced regulation of NOX gene expression (Figure 12) and protein synthesis (Figure 13) in INS-1E. Transcription of *Nox1* and *Duox1* is transiently upregulated at early periods (4–8 h), returning to baseline at 16 h. Transcription of *Nox2* and *p47phox* is also upregulated at early periods, but elevated expression persists until 24 h. In contrast, transcription of *Duox(a)2*, *p67phox* and *p40phox* is downregulated at later periods (16–24 h). However, no significant changes were observed in protein synthesis, except for a modest late (24 h) upregulation of DUOX1 (Figure 13). This discrepancy may be due to the smaller number of independent experiments (N = 3) and/or antibody off-targeting [45]. Nevertheless, our RT-qPCR data are consistent with previous studies. Cytokines induced early and transient upregulation of *Nox1* transcription in INS-1 [30] and early and sustained upregulation of p47phox protein synthesis in BRIN-BD11 [26]. Our data also corroborate the notoriously low (C_T_ > 30) *NOX1* mRNA expression levels reported in human islets [18,30]. In untreated INS1E cells, *Nox1* and *Duox1* had the lowest transcript levels among NOX isoforms (Additional File 6: Treatment). While these isoforms may have minimal expression under basal conditions, their transcription might be transiently increased by inflammation (Figure 12) [31] and during Type 2 Diabetes *mellitus* [30].

In conclusion, the isoform-specific, site-specific and time-dependent function of NOXs in β cell (dys)function merits further attention. Particularly the relative contribution of each isoform to ROS production in response to glucose and cytokines, as well as their possible role in regulating Ca^2+^ signaling and SNARE dynamics. Likewise, the downstream targets of NOX activity during cytokine-induced β cell death ought future research – ER stress and NF-κB signaling being especially relevant in this context. Such studies might reveal NOXs as proximal fine tuners of early signaling events in β cell (patho)physiology, instead of distal effectors of unspecific and unrestricted cell damage as traditionally thought.

## Supplementary Material

Supplemental Material

## Data Availability

The authors confirm that the data supporting the findings of this study are available within the article and its supplementary materials; except for immunofluorescence data, openly available at BioImage Archive.

## Data deposition

Immunofluorescence data that support the findings of this study are openly available in BioImage Archive at https://doi.org/10.6019/S-BIAD1502, reference number S-BIAD1502.
