# Supplementary material for: Expression, localization and regulation of NADPH oxidases in pancreatic beta cells
Source: Redox Rep. 2025 Oct 6;30(1):2568300. doi: 10.1080/13510002.2025.2568300 (PMC12507117; doi:10.1080/13510002.2025.2568300)
Supplement: Supplemental Material [file YRER_A_2568300_SM5385.zip › YRER_A_2568300_Supplement file/Additional File 1 Material.pdf]

Additional File 1 – Material

Expression, Localization and Regulation of NADPH Oxidases in Pancreatic Beta Cells

Davidson Correa de Almeida, Eloisa Aparecida Vilas-Boas, Paulo Henrique Coelho Ferreira, Sandra Mara Ferreira, Angelo Rafael Carpinelli, Fernanda Ortis

Supplementary Table S1 – Material

**Material (Manufacturer)**	**Manufacturer / Supplier**	**Catalog**
40% Acrylamide/Bis Solution, 37.5:1	Bio-Rad Laboratories	1610148
Clarity Western ECL Substrate, 500 ml	Bio-Rad Laboratories	1705060
Ethylenediaminetetraacetic Acid	Bio-Rad Laboratories	1610729
Glicine	Bio-Rad Laboratories	1610724
Nitrocellulose Membrane	Bio-Rad Laboratories	1620112
Precision Plus Protein Dual Color Standards	Bio-Rad Laboratories	1610374
Sodium Dodecyl Sulfate	Bio-Rad Laboratories	1610302
TEMED	Bio-Rad Laboratories	1610801
Tris base	Bio-Rad Laboratories	1610716
Triton X-100	Bio-Rad Laboratories	161-0407
Tween 20	Bio-Rad Laboratories	1706531
Acetic Acid	Dinâmina Química Analítica	N/A
Boric Acid	Dinâmina Química Analítica	N/A
Primers	Exxtend Biotecnologia	N/A
Sucrose (União)	Grupo Camil	N/A
Microscope Slides 26x76x01 mm (Olen)	Kasvi	K5-7102
Rectangular coverslips 24x50 mm (0.13-0.16 mm) (Olen)	Kasvi	K5-2450
CaCl2-2H2O	Labsynth	N/A
Citric Acid	Labsynth	N/A
D-glucose	Labsynth	N/A
Glycerol	Labsynth	N/A
HCl	Labsynth	N/A
KCl	Labsynth	N/A
KH2PO4	Labsynth	N/A
MgSO4-7H2O	Labsynth	N/A
Na2PO4-12H2O	Labsynth	N/A
NaCl	Labsynth	N/A
NaHCO3	Labsynth	N/A
Paraformaldehyde	Labsynth	N/A
Sodium Bicarbonate	Labsynth	N/A
Xylene	Labsynth	N/A
WesternSure Pen	LI-COR	926-91000
2-mercaptoethanol	Merck-Millipore	8057400250
Non-fat Dry Milk (Molico)	Nestlé	N/A
Gel Loading Dye Purple 6X	New England BioLabs	N0556S
Quick-Load Purple 50 bp DNA Ladder	New England BioLabs	N0556S
Ammonium Persulfate	Sigma-Aldrich	A3678
Bovine Serum Albumin	Sigma-Aldrich	A4503
Collagenase from Clostridium histolyticum Type V	Sigma-Aldrich	C9263
HEPES	Sigma-Aldrich	H3375
Phosphate Saline Buffer tablet	Sigma-Aldrich	P4417
Poly-L-lysine solution	Sigma-Aldrich	P4832

Ponceau S	Sigma-Aldrich	P3504
Sodium Deoxycholate	Sigma-Aldrich	D6750
Sodium Pyruvate	Sigma-Aldrich	P2256
Thapsigargin	Sigma-Aldrich	T9033
Microscope slides 26x76x01 mm (Knittel)	SPLab	VS111711FKB.01
Round coverslips 13mm (0,13-0,17 mm) (Knittel)	SPLab	VD10013Y1A.01
50 bp DNA Ladder	ThermoFisher Scientific	10416014
Alexa Fluor 488 Phalloidin	ThermoFisher Scientific	A12379
ezDNase Enzyme	ThermoFisher Scientific	11766051
Fetal Bovine Serum, qualified, Brazil	ThermoFisher Scientific	12657029
Glycogen	ThermoFisher Scientific	AM9510
Halt Protease Inhibitor Cocktail	ThermoFisher Scientific	87786
Millennium RNA Markers	ThermoFisher Scientific	AM7150
Penicillin-Streptomycin 10,000 U/mL	ThermoFisher Scientific	15140122
Pierce BCA Protein Assay Kit	ThermoFisher Scientific	23227
PowerUp SYBR Green Master Mix	ThermoFisher Scientific	A25776
ProLong Glass Antifade Mountant with NucBlue Stain	ThermoFisher Scientific	P36981
Rat IFN-gamma Recombinant Protein	ThermoFisher Scientific	RP-8616
Rat IL-1 beta Recombinant Protein	ThermoFisher Scientific	RP-87923
Rat TNF-alpha Recombinant Protein	ThermoFisher Scientific	RP-87939
RPMI 1640 Medium	ThermoFisher Scientific	31800022
SuperScript IV VILO Master Mix with ezDNase Enzyme	ThermoFisher Scientific	11766050
TRIzol Reagent	ThermoFisher Scientific	15596018
Agarose (Kasvi)	UniScience	K9-9500
GelRed 10.000X in water - 0,5 ML (Biotium)	UniScience	41003
Bromophenol Blue Sodium Salt	USB	US12370

Supplementary Table S2 – Primers

Gene	Sense	5'-3' sequence	Tm	Amplicon (bp)
Rn18s	Forward	TTCCCAGTAAGTGCGGGTCAT	61.45	167
	Reverse	AGTCAAGTTCGACCGTCTTCTCA	61.55	
Gapdh	Forward	AGTGCCAGCCTCGTCTCATA	60.68	92
	Reverse	AGAGAAGGCAGCCCTGGTAA	60.55	
Duox1#1*	Forward	TGTGCAAGATTTTTGGCCCG	59.97	166
	Reverse	CGAGAGTGCAGGGTTGATGT	60.04	
Duox2#1*	Forward	GACTCCTGGGAACAGGGTAATG	60.09	83
	Reverse	CTAGGAAGCCCCTCTGCATTT	59.79	
Duoxa1#1*	Forward	CCCACACAGTCGGGGCTCTA	63.42	178
	Reverse	TCAGATACTGTGCGCGTGGC	63.16	
Duoxa1#2*	Forward	AAGATAAACGGGGTGCGGG	60.08	144
	Reverse	GAACCGGGTCCAGACGTAGA	60.96	
Duoxa2#1*	Forward	CCTTCAGTACCTCCCGAGTTCA	61.15	87
	Reverse	GCATTGGTGTTCCTCTGAGTGT	60.81	
Atf3	Forward	TGCTGCTGCCAAGTGTCGAA	62.94	181
	Reverse	CTGAGCCCGGACGATACACG	62.66	
Chop	Forward	TGAGTCTCTGCCTTTCGCCTT	61.71	177
	Reverse	CAAGCTAGGGATGCAGGGTCA	61.86	
Cxcl10	Forward	CATCCCGAGCCAACCTTCCA	62.2	112
	Reverse	CACCGTTCTTGCGAGAGGGA	62.15	

Nox1	Forward	ATGAGTGAAAGTCATCCCCGC	60.41	112
	Reverse	ACTTCCAAGACTCAGGGGGTT	60.7	
Nox2	Forward	TGCCCAGTACCAAAGTTTGCC	61.37	58
	Reverse	GACCCACGATCCATTTCCAAG	58.98	
Nox4	Forward	CCACTTGGTGAACGCCCTGA	62.67	143
	Reverse	CCACCACCATGCAGACACCT	62.42	
Duox1#2**	Forward	TTGGTCGCCCTCCCTTTGAG	62.12	197
	Reverse	AGTGGGCATAGTTGCTGGCT	61.86	
Duox2#2**	Forward	TCCATTTCATGCTGCGCGAC	61.98	163
	Reverse	CGGGGAGAGGGAGCCATTAC	61.76	
Duoxa2#2**	Forward	CACCGAACAGTCCTTGCGGA	62.71	199
	Reverse	GAGATCGAGGCGAAGGCGAA	62.34	
p67phox	Forward	TACCTGACGGCCAAAGATGG	59.75	97
	Reverse	AACCCTTCTCGTAGTCAGCG	59.47	
p47phox	Forward	CGAATTCACCCTCAGTCGCA	60.39	93
	Reverse	CTGGAGTCTTTCTGGGGGAC	59.38	
p40phox	Forward	CAAGCTGGAGGAGCGGTTTG	61.58	139
	Reverse	CATGTAGGCATTGAGGGCCG	61.17	
p22phox	Forward	CCGGGCTGTCCTCCACTTAC	62.24	198
	Reverse	GGGTTGGTAGGTGGCTGCTT	62.43	
Noxa1	Forward	CTCGGCAAGGCTAAGGTGGT	62.19	139
	Reverse	CCCTTGTGCTCAGGACCCTC	62.19	
Noxo1	Forward	GGAGCTGGGAGGAGTTTAGGC	62.19	200
	Reverse	TGAGGTTGCCAGCAGTGACC	62.68	
Rac1	Forward	TCTCCTACCCGCAAACAGAC	59.39	148
	Reverse	AGATCAAGCTTCGTCCCCAC	59.75	

*Used in Fig. 1-3. **Used in Fig. 9.

Supplementary Table S3 – Antibodies

Antibody	Host / clonality	Conjugation	IF	WB	Manufacturer	Catalog
NOX1	Rabbit pAb	N/A	1:200	1:1000	Novus Biologicals	NBP1-31546
NOX2	Rabbit pAb	N/A	1:100	1:500	Bioss Antibodies	bs-3889R
NOX4	Rabbit pAb	N/A	1:100	1:1000	Novus Biologicals	NB110-58851
DUOX1	Rabbit pAb	N/A	1:100	1:1000	Bioss Antibodies	bs-11431R
DUOX2	Rabbit pAb	N/A	1:100	1:500	Novus Biologicals	NB110-61576
P-eIF2a	Rabbit pAb	N/A	N/A	1:1000	Cell Signaling Technology	#9721
Caspase 3	Rabbit pAb	N/A	N/A	1:1000	Cell Signaling Technology	#9662
α-tubulin	Mouse mAb	N/A	N/A	1:1000	invitrogen	#32-2500
Rabbit IgG	Goat	HRP	N/A	1:10000	Bio-Rad Laboratories	#170-5046
Mouse IgG	Goat	HRP	N/A	1:10000	Bio-Rad Laboratories	#170-5047
Insulin	Mouse mAb	AF488	1:100	N/A	invitrogen	#53-9769-82
PDI	Mouse mAb	N/A	1:100	N/A	invitrogen	MA3-019
Syntaxin 1	Mouse mAb	N/A	1:100	N/A	invitrogen	MA5-17612
LAMP1	Mouse mAb	N/A	1:100	N/A	invitrogen	MA1-164
Rabbit IgG	Goat pAb	AF568	1:500	N/A	invitrogen	A-11036
Mouse IgG	Goat pAb	AF488	1:500	N/A	invitrogen	A-11001

pAb: polyclonal antibody, mAb: monoclonal antibody.

```
//ImageJ Macro 1 – IHC Image Processing (.ijm)

#@ File (label = "Input directory", style = "directory") input
#@ File (label = "Processed directory", style = "directory") output
#@ String (label = "File suffix", value = ".czi") suffix

setBatchMode(true);
processFolder(input);
print("\\Clear");
print("Done!");

function processFolder(input) {
	list = getFileList(input);
	list = Array.sort(list);
	for (i = 0; i < list.length; i++) {
		if(File.isDirectory(input + File.separator + list[i]))
			processFolder(input + File.separator + list[i]);
		if(endsWith(list[i], suffix))
			processFile(input, output, list[i]);
	}
}

function processFile(input, output, file) {
	inputPath = input + File.separator + list[i];
	print("Processing: " + file);
	run("Bio-Formats Importer", "open=[" + inputPath + "] color_mode=Default
rois_import=[ROI manager] view=Hyperstack stack_order=XYCZT");

	name = getTitle();
	run("Split Channels");

	selectImage("C1-" + name);
	dapi = getTitle();
		run("Subtract Background...", "rolling=5 sliding disable");
		run("Enhance Contrast...", "saturated=0.1 normalize");

	selectImage("C2-" + name);
	fitc = getTitle();
		run("Subtract Background...", "rolling=5 sliding disable");
		run("Enhance Contrast...", "saturated=0.1 normalize");

	selectImage("C3-" + name);
	rhoda = getTitle();
		run("Subtract Background...", "rolling=5 sliding disable");

	run("Merge Channels...", "c1=[" + dapi + "] c2=[" + fitc + "] c3=[" + rhoda + "]
create");

	fileName = File.nameWithoutExtension;
	fileName = replace(fileName, " 20x", "");
	fileName = replace(fileName, "-S", " S");
	savePath = output + File.separator + fileName;
	run("Bio-Formats Exporter", "save=[" + savePath + ".ome.tif]
compression=Uncompressed");
	close("*");

}

// end of Macro 1

// ImageJ Macro 2 – IHC Measure px Intensity (.ijm)

#@ File (label = "Input directory", style = "directory") input
#@ File (label = "Results directory", style = "directory") output
#@ File (label = "Mask directory", style = "directory") output2
#@ String (label = "File suffix", value = ".czi") suffix
```

```
setBatchMode(true);
processFolder(input);
selectWindow("Results");
saveAs("Text", output + File.separator + "Pixel-Intensity_Results.csv");
print("\\Clear");
print("Done!");

function processFolder(input) {
	list = getFileList(input);
	list = Array.sort(list);
	for (i = 0; i < list.length; i++) {
		if(File.isDirectory(input + File.separator + list[i]))
			processFolder(input + File.separator + list[i]);
		if(endsWith(list[i], suffix))
			processFile(input, output, list[i]);
	}
}

function processFile(input, output, file) {
	inputPath = input + File.separator + list[i];
	print("Processing: " + file);
	bur  =  "open=["  +  inputPath  +  "]  color_mode=Default  rois_import=[ROI  manager]
view=Hyperstack stack_order=XYCZT";
	run("Bio-Formats Importer", bur);

	name = getTitle();
	run("Split Channels");

	selectImage("C1-" + name);
	dapi = getTitle();
	selectImage("C3-" + name);
	rhoda = getTitle();
	selectImage("C2-" + name);
	fitc = getTitle();

	run("Duplicate...", " ");
	mask = getTitle();
	setAutoThreshold("Huang dark");
	//run("Threshold...");
	run("Convert to Mask");
	run("Open");
	run("Close-");
	run("Median", "radius=3");
	run("Create Selection");

	selectImage(rhoda);
	run("Restore Selection");
	run("Set Measurements...", "area mean standard modal min integrated median display
redirect=None decimal=4");
	run("Measure");
	run("Make Inverse");
	run("Measure");

	selectImage(mask);
	run("Select None");
	run("16-bit");
	run("Invert");

	run("Merge Channels...", "c1=[" + dapi + "] c2=[" + fitc + "] c3=[" + rhoda + "]
c4=[" + mask + "] create keep");

	fileName = File.nameWithoutExtension;
	fileName = replace(fileName, "_ome", "_mask");
	savePath = output2 + File.separator + fileName;
```

```
	run("Bio-Formats Exporter", "save=[" + savePath + ".ome.tif] compression=Uncompressed");

	selectImage(fitc);
	run("Duplicate...", "title=holes");
	holes = getTitle();
	setAutoThreshold("Huang dark");
	//run("Threshold...");
	run("Convert to Mask");
	run("Open");
	run("Close-");
	run("Median", "radius=3");

	run("Duplicate...", " ");
	full = getTitle();
	run("Fill Holes");
	run("Create Selection");
	run("Make Inverse");

	selectImage(holes);
	run("Restore Selection");
	run("Add...", "value=255");
	run("Select None");
	run("Convert to Mask");
	run("Create Selection");

	selectImage(rhoda);
	run("Restore Selection");
	run("Measure");

	run("Close All");

}

// end of Macro 2

// ImageJ Macro 3 – ICC Image Processing and Measure px Intensity (.ijm)

#@ File (label = "Input directory", style = "directory") input
#@ File (label = "Results directory", style = "directory") output
#@ File (label = "Corrected directory", style = "directory") output2
#@ File (label = "Mask directory", style = "directory") output3
#@ String (label = "File suffix", value = ".czi") suffix

setBatchMode(true);
processFolder(input);
selectWindow("Results");
saveAs("Text", output + File.separator + "Pixel-Intensity_Results.csv");
print("\\Clear");
print("Done!");

function processFolder(input) {
	list = getFileList(input);
	list = Array.sort(list);
	for (i = 0; i < list.length; i++) {
		if(File.isDirectory(input + File.separator + list[i]))
			processFolder(input + File.separator + list[i]);
		if(endsWith(list[i], suffix))
			processFile(input, output, list[i]);
	}
}

function processFile(input, output, file) {
	inputPath = input + File.separator + list[i];
	print("Processing: " + file);
```

```
	bur = "open=[" + inputPath + "] color_mode=Default rois_import=[ROI manager] view=Hyperstack stack_order=XYCZT";
	run("Bio-Formats Importer", bur);

	name = getTitle();
	run("Split Channels");

	selectImage("C1-" + name);
	dapi = getTitle();
	run("Duplicate...", " ");
	setAutoThreshold("Huang dark");
	//run("Threshold...");
	run("Convert to Mask");
	run("Open");
	run("Close-");
	run("Median", "radius=3");
	dapiMask = getTitle();

	selectImage(dapi);
	run("Subtract Background...", "rolling=5 sliding disable");
	run("Enhance Contrast...", "saturated=0.1 normalize");

	selectImage("C2-" + name);
	fitc = getTitle();
	run("Duplicate...", " ");
	setAutoThreshold("Huang dark");
	//run("Threshold...");
	run("Convert to Mask");
	run("Open");
	run("Close-");
	run("Median", "radius=3");
	fitcMask = getTitle();

	selectImage(fitc);
	run("Subtract Background...", "rolling=5 sliding disable");
	run("Enhance Contrast...", "saturated=0.1 normalize");

	selectImage("C3-" + name);
	rhoda = getTitle();
	run("Duplicate...", " ");
	setAutoThreshold("Huang dark");
	//run("Threshold...");
	run("Convert to Mask");
	run("Open");
	run("Close-");
	run("Median", "radius=3");
	rhodaMask = getTitle();

	selectImage(rhoda);
	run("Subtract Background...", "rolling=5 sliding disable");

	run("Merge Channels...", "c1=[" + dapiMask + "] c2=[" + fitcMask + "] c3=[" + rhodaMask + "] create");
	compositeMask = getTitle();
	run("Z Project...", "projection=[Max Intensity]");
	mask = getTitle();
	run("Create Selection");
	run("Make Inverse");

	selectImage(rhoda);
	run("Restore Selection");
	run("Set Measurements...", "area mean standard modal min integrated median display redirect=None decimal=4");
	run("Measure");
	run("Make Inverse");
	run("Measure");
```

```
        selectImage(mask);
        run("Select None");
        run("16-bit");
        run("Invert");

        run("Merge Channels...", "c1=[" + dapi + "] c2=[" + rhoda + "] create keep");
        fileName = File.nameWithoutExtension;
        savePath = output2 + File.separator + fileName;
        run("Bio-Formats     Exporter",     "save=["     +     savePath     +     ".ome.tif]
compression=Uncompressed");

        run("Merge Channels...", "c1=[" + dapi + "] c2=[" + rhoda + "] c3=[" + mask + "]
create keep");
        fileName = File.nameWithoutExtension;
        savePath = output3 + File.separator + fileName;
        run("Bio-Formats    Exporter",    "save=["    +    savePath    +    "_mask.ome.tif]
compression=Uncompressed");

        run("Close All");

}

// end of Macro 3

// ImageJ Macro 4 - ICC Image Processing for Association Analysis (.ijm)

#@ File (label = "Input directory", style = "directory") input
#@ File (label = "Processed directory", style = "directory") output
#@ String (label = "File suffix", value = ".czi") suffix

setBatchMode(true);
processFolder(input);
print("\\Clear");
print("Done!");

function processFolder(input) {
        list = getFileList(input);
        list = Array.sort(list);
        for (i = 0; i < list.length; i++) {
                if(File.isDirectory(input + File.separator + list[i]))
                        processFolder(input + File.separator + list[i]);
                if(endsWith(list[i], suffix))
                        processFile(input, output, list[i]);
        }
}

function processFile(input, output, file) {
        inputPath = input + File.separator + list[i];
        print("Processing: " + file);
        run("Bio-Formats   Importer",   "open=["   +   inputPath   +   "]   color_mode=Default
rois_import=[ROI manager] view=Hyperstack stack_order=XYCZT");

        name = getTitle();
        run("Split Channels");

        selectImage("C1-" + name);
        dapi = getTitle();
        run("Smooth", "stack");
        run("Subtract Background...", "rolling=5 sliding disable stack");
        run("Enhance Contrast...", "saturated=0.1 process_all use");

        selectImage("C2-" + name);
        fitc = getTitle();
        run("Smooth", "stack");
        run("Subtract Background...", "rolling=5 sliding disable stack");
```

```
	run("Enhance Contrast...", "saturated=0.1 process_all use");

	selectImage("C3-" + name);
	rhoda = getTitle();
	run("Smooth", "stack");
	run("Subtract Background...", "rolling=5 sliding disable stack");
	run("Enhance Contrast...", "saturated=0.1 process_all use");

	run("Merge Channels...", "c1=[" + dapi + "] c2=[" + fitc + "] c3=[" + rhoda + "]
create");

	fileName = File.nameWithoutExtension;
	savePath = output + File.separator + fileName;
	run("Bio-Formats     Exporter",     "save=["     +     savePath     +     ".ome.tif]
compression=Uncompressed");
	close("*");

}

// end of Macro 4

// ImageJ Macro 5 – ICC Association Analysis (.ijm)

#@ File (label = "Input directory", style = "directory") input
#@ File (label = "Results directory", style = "directory") output
#@ File (label = "Mask directory", style = "directory") output2
#@ String (label = "File suffix", value = ".czi") suffix

setBatchMode(true);
processFolder(input);
selectWindow("Results");
saveAs("Text", output + File.separator + "BIOP-JACoP_Results.csv");
print("\\Clear");
print("Done!");

function processFolder(input) {
	list = getFileList(input);
	list = Array.sort(list);
	for (i = 0; i < list.length; i++) {
		if(File.isDirectory(input + File.separator + list[i]))
			processFolder(input + File.separator + list[i]);
		if(endsWith(list[i], suffix))
			processFile(input, output, list[i]);
	}
}

function processFile(input, output, file) {
	inputPath = input + File.separator + list[i];
	bur  =  "open=["  +  inputPath  +  "]  color_mode=Default  rois_import=[ROI  manager]
view=Hyperstack stack_order=XYCZT";
	run("Bio-Formats Importer", bur);

	name = getTitle();
	run("Split Channels");

	selectImage("C1-" + name);
	dapi = getTitle();
	run("Z Project...", "projection=[Max Intensity]");
	setAutoThreshold("Huang dark");
	//run("Threshold...");
	run("Convert to Mask");
	dapiMask = getTitle();

	selectImage("C2-" + name);
	fitc = getTitle();
	run("Z Project...", "projection=[Max Intensity]");
```

```
	setAutoThreshold("Huang dark");
	//run("Threshold...");
	run("Convert to Mask");
	fitcMask = getTitle();

	selectImage("C3-" + name);
	rhoda = getTitle();
	run("Z Project...", "projection=[Max Intensity]");
	setAutoThreshold("Huang dark");
	//run("Threshold...");
	run("Convert to Mask");
	rhodaMask = getTitle();

	run("Merge Channels...", "c1=[" + dapiMask + "] c2=[" + fitcMask + "] c3=[" +
rhodaMask + "] create");
	compositeMask = getTitle();

	fileName = File.nameWithoutExtension;
	fileName = replace(fileName, "_ome", "_Mask");
	savePath = output2 + File.separator + fileName;
	run("Bio-Formats Exporter", "save=[" + savePath + ".ome.tif]
compression=Uncompressed");

	run("Z Project...", "projection=[Max Intensity]");
	mask = getTitle();
	selectImage(mask);
	run("Create Selection");
	run("Make Inverse");

	selectImage(dapi);
	run("Merge Channels...", "c1=[" + dapi + "] c2=[" + fitc + "] c3=[" + rhoda + "]
create");
	composite = getTitle();

	selectImage(mask);
	selectImage(composite);
	run("Restore Selection");

	run("BIOP JACoP", "channel_a=3 channel_b=2 threshold_for_channel_a=Otsu
threshold_for_channel_b=Otsu manual_threshold_a=0 manual_threshold_b=0 crop_rois
get_pearsons get_manders costes_block_size=5 costes_number_of_shuffling=100");
	selectImage(composite);
	run("BIOP JACoP", "channel_a=3 channel_b=1 threshold_for_channel_a=Otsu
threshold_for_channel_b=Otsu manual_threshold_a=0 manual_threshold_b=0 crop_rois
get_pearsons get_manders costes_block_size=5 costes_number_of_shuffling=100");
	selectImage(composite);
	run("BIOP JACoP", "channel_a=2 channel_b=1 threshold_for_channel_a=Otsu
threshold_for_channel_b=Otsu manual_threshold_a=0 manual_threshold_b=0 crop_rois
get_pearsons get_manders costes_block_size=5 costes_number_of_shuffling=100");
	run("Close All");

}

// end of Macro 5

// ImageJ Macro 6 - CLSM Image Processing (.ijm)

#@ File (label = "Input directory", style = "directory") input
#@ File (label = "Processed directory", style = "directory") output
#@ String (label = "File suffix", value = ".czi") suffix

setBatchMode(true);
processFolder(input);
print("\\Clear");
print("Done!");
```

```
function processFolder(input) {
	list = getFileList(input);
	list = Array.sort(list);
	for (i = 0; i < list.length; i++) {
		if(File.isDirectory(input + File.separator + list[i]))
			processFolder(input + File.separator + list[i]);
		if(endsWith(list[i], suffix))
			processFile(input, output, list[i]);
	}
}

function processFile(input, output, file) {
	inputPath = input + File.separator + list[i];
	print("Processing: " + file);
	run("Bio-Formats Importer", "open=[" + inputPath + "] color_mode=Default
rois_import=[ROI manager] view=Hyperstack stack_order=XYCZT");

	name = getTitle();
	run("Split Channels");

	selectImage("C1-" + name);
	dapi = getTitle();
	run("Subtract Background...", "rolling=5 sliding disable stack");
	run("Enhance Contrast...", "saturated=0.1 process_all use");

	selectImage("C2-" + name);
	fitc = getTitle();
	run("Subtract Background...", "rolling=5 sliding disable stack");
	run("Enhance Contrast...", "saturated=0.1 process_all use");

	selectImage("C3-" + name);
	rhoda = getTitle();
	run("Subtract Background...", "rolling=5 sliding disable stack");
	run("Enhance Contrast...", "saturated=0.1 process_all use");

	run("Merge Channels...", "c1=[" + dapi + "] c2=[" + fitc + "] c3=[" + rhoda + "]
create");

	fileName = File.nameWithoutExtension;
	fileName = replace(fileName, "ome-", "");
	savePath = output + File.separator + fileName;
	run("Bio-Formats Exporter", "save=[" + savePath + ".ome.tif]
compression=Uncompressed");
	close("*");

}

// end of Macro 6

// ImageJ Macro 7 - CLSM Association Analysis (.ijm)

#@ File (label = "Input directory", style = "directory") input
#@ File (label = "Results directory", style = "directory") output
#@ File (label = "Mask directory", style = "directory") output2
#@ String (label = "File suffix", value = ".czi") suffix

setBatchMode(true);
processFolder(input);
selectWindow("Results");
saveAs("Text", output + File.separator + "BIOP-JACoP_Results.csv");
print("\\Clear");
print("Done!");

function processFolder(input) {
	list = getFileList(input);
	list = Array.sort(list);
```

```
	for (i = 0; i < list.length; i++) {
		if(File.isDirectory(input + File.separator + list[i]))
			processFolder(input + File.separator + list[i]);
		if(endsWith(list[i], suffix))
			processFile(input, output, list[i]);
	}
}

function processFile(input, output, file) {
	inputPath = input + File.separator + list[i];
	bur = "open=[" + inputPath + "] color_mode=Default rois_import=[ROI manager]
view=Hyperstack stack_order=XYCZT";
	run("Bio-Formats Importer", bur);

	name = getTitle();
	run("Split Channels");

	selectImage("C1-" + name);
	dapi = getTitle();
	run("Z Project...", "projection=[Max Intensity]");
	setAutoThreshold("Huang dark");
	//run("Threshold...");
	run("Convert to Mask");
	dapiMask = getTitle();

	selectImage("C2-" + name);
	fitc = getTitle();
	run("Z Project...", "projection=[Max Intensity]");
	setAutoThreshold("Huang dark");
	//run("Threshold...");
	run("Convert to Mask");
	fitcMask = getTitle();

	selectImage("C3-" + name);
	rhoda = getTitle();
	run("Z Project...", "projection=[Max Intensity]");
	setAutoThreshold("Huang dark");
	//run("Threshold...");
	run("Convert to Mask");
	rhodaMask = getTitle();

	run("Merge Channels...", "c1=[" + dapiMask + "] c2=[" + fitcMask + "] c3=[" +
rhodaMask + "] create");
	compositeMask = getTitle();

	fileName = File.nameWithoutExtension;
	fileName = replace(fileName, "_ome", "_Mask");
	savePath = output2 + File.separator + fileName;
	run("Bio-Formats Exporter", "save=[" + savePath + ".ome.tif]
compression=Uncompressed");

	run("Z Project...", "projection=[Max Intensity]");
	mask = getTitle();
	selectImage(mask);
	run("Create Selection");
	run("Make Inverse");

	selectImage(dapi);
	run("Merge Channels...", "c1=[" + dapi + "] c2=[" + fitc + "] c3=[" + rhoda + "]
create");
	composite = getTitle();

	selectImage(mask);
	selectImage(composite);
	run("Restore Selection");
```

```
	run("BIOP JACoP", "channel_a=3 channel_b=2 threshold_for_channel_a=Otsu threshold_for_channel_b=Otsu manual_threshold_a=0 manual_threshold_b=0 crop_rois get_pearsons get_manders costes_block_size=5 costes_number_of_shuffling=100");
	selectImage(composite);
	run("BIOP JACoP", "channel_a=3 channel_b=1 threshold_for_channel_a=Otsu threshold_for_channel_b=Otsu manual_threshold_a=0 manual_threshold_b=0 crop_rois get_pearsons get_manders costes_block_size=5 costes_number_of_shuffling=100");
	selectImage(composite);
	run("BIOP JACoP", "channel_a=2 channel_b=1 threshold_for_channel_a=Otsu threshold_for_channel_b=Otsu manual_threshold_a=0 manual_threshold_b=0 crop_rois get_pearsons get_manders costes_block_size=5 costes_number_of_shuffling=100");
	run("Close All");

}

// end of Macro 7
```
