# Supplementary material for: Expression, localization and regulation of NADPH oxidases in pancreatic beta cells
Source: Redox Rep. 2025 Oct 6;30(1):2568300. doi: 10.1080/13510002.2025.2568300 (PMC12507117; doi:10.1080/13510002.2025.2568300)
Supplement: Supplemental Material [file YRER_A_2568300_SM5385.zip › YRER_A_2568300_Supplement file/Additional File 7 Supplementary Figures.pdf]

Supplementary Figure S1 – Dissociation Curves

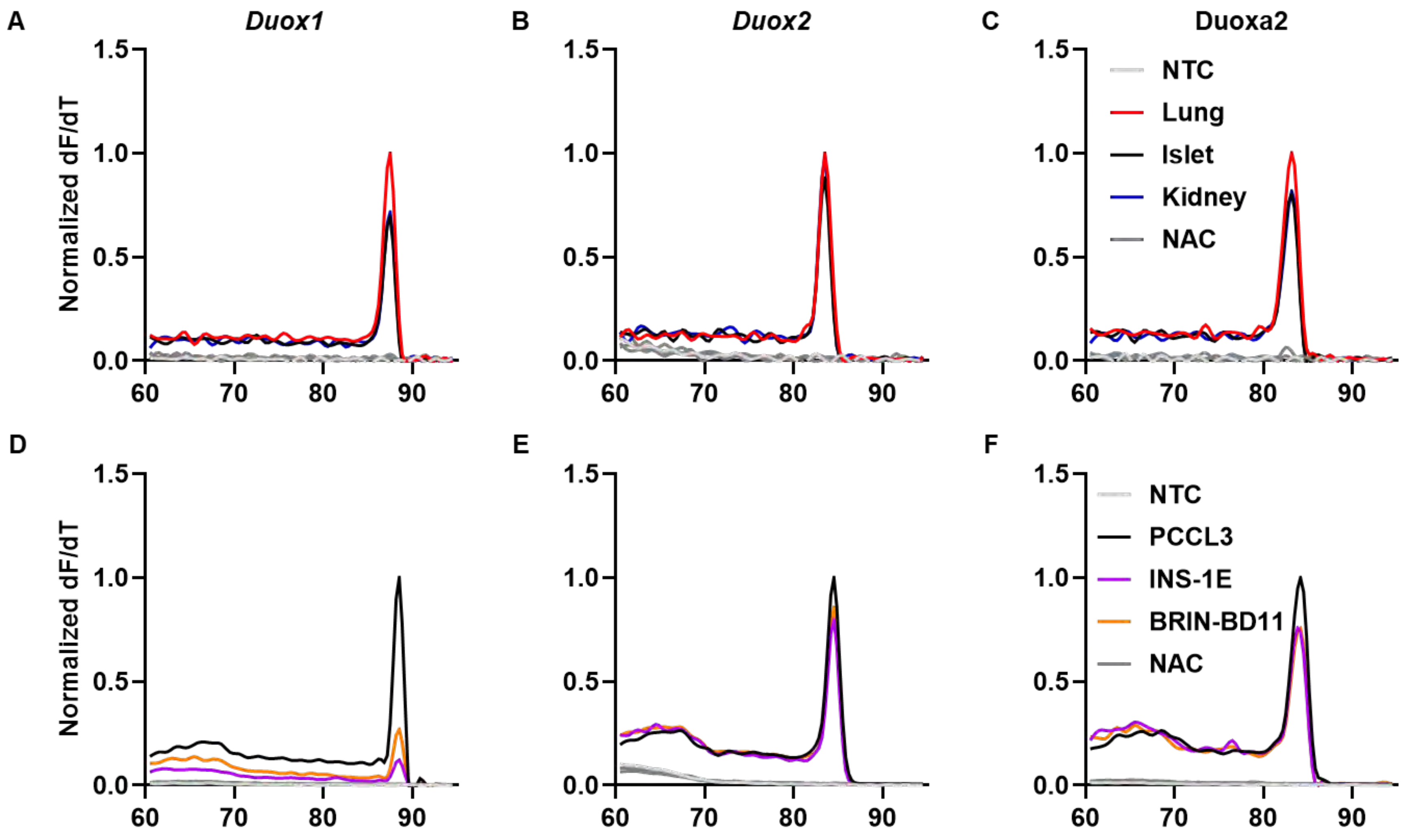

Supplementary Figure S1. Dissociation curves for *Duox1* and *Duox(a)2*.

Expression of *Duox1* (A and D), *Duox2* (B and E) and *Duoxa2* (C and F) in rat pancreatic islets (A-C) and two rat β cell lines (INS-1E and BRIN-BD11) (D-F) was measured by RT-qPCR. Lungs and the thyroid cell line PCCL3 were used as positive amplification controls for tissues and cell lines, respectively; kidneys were used as negative amplification controls for tissues. Dissociation curves for the indicated genes are shown. Each plotted line represents the mean of 4-8 biological replicates in technical triplicates each. For each gene, dF/dT was normalized by the average peak value of lungs (A-C) or PCCL3 (D-F). NTC: no template control, NAC: no amplification control. Gray: NTC and NAC, black: islets (A-C) or PCCL3 (D-F), red: lungs, blue: kidneys, purple: INS-1E, orange: BRIN-BD11.

Supplementary Figure S2 – Gels in Fig. 1-2

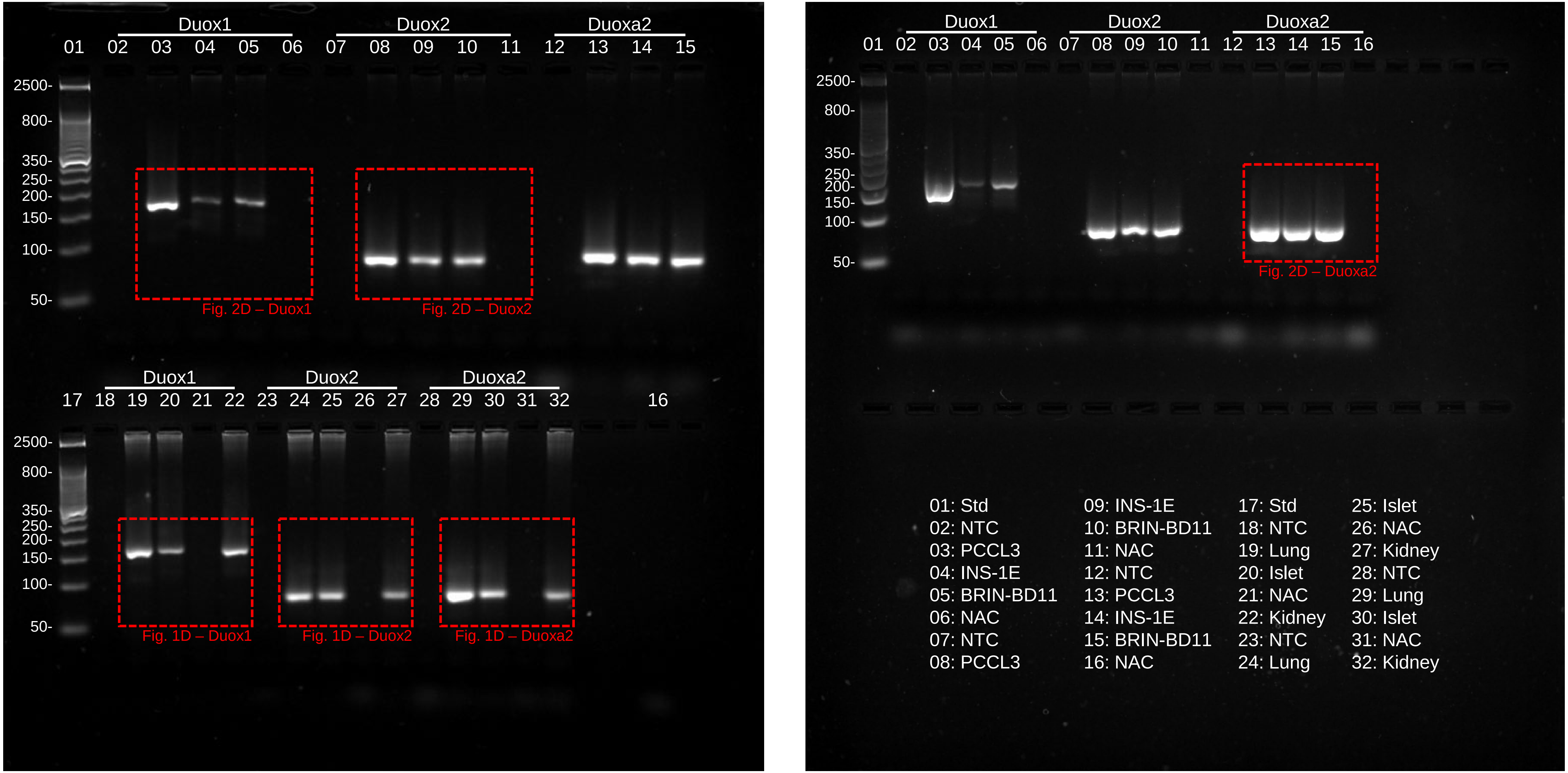

Supplementary Figure S2. Uncropped gels in Fig. 1A and Fig. 2A.

Two uncropped gels are shown. Samples from cell lines in the two gels are biological replicates of one another. Genes and samples are indicated at the top and the molecular weight in bp to the left. Std: Invitrogen 50 bp DNA Ladder, NTC: no template control, NAC: no amplification control (from islets or INS-1E). Red boxes: insets in Fig. 1A and Fig. 2A.

Supplementary Figure S3 – *Duoxa1* Dissociation Curves

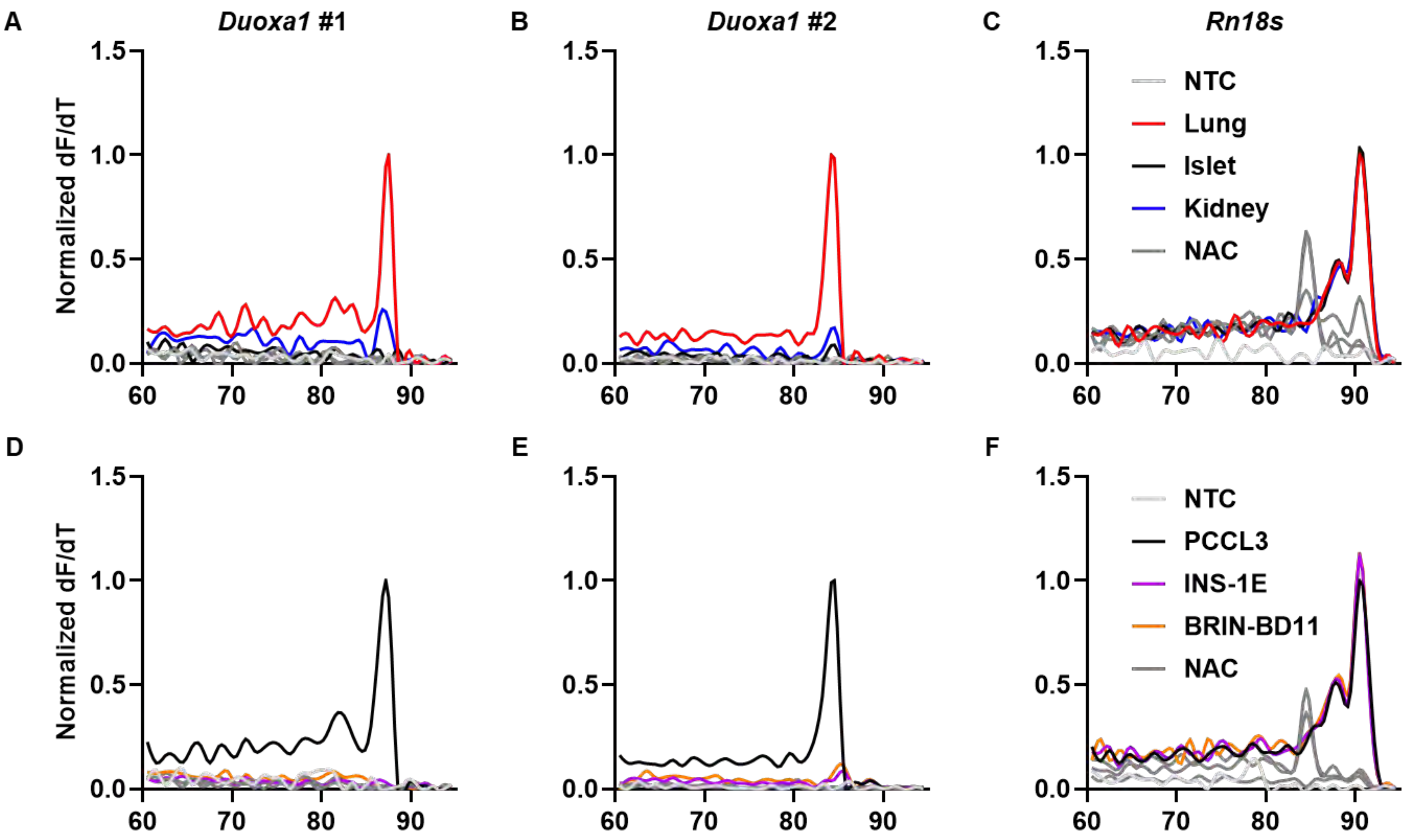

Supplementary Figure S3. Dissociation curves for *Duox1* and *Duox(a)2*.

Expression of *Duoxa1* in rat pancreatic islets (A-C) and two rat β cell lines (INS-1E and BRIN-BD11) (D-F) was analysed by RT-qPCR with two sets of primers: *Duoxa1#1* (A and D) and *Duoxa1#2* (B and E). The *Rn18s* gene (C and F) was used as a positive amplification control; as well as lungs and the thyroid cell line PCCL3 (for tissues and cell lines, respectively). Kidneys were used as negative amplification controls for tissues. Dissociation curves for the indicated genes are shown. Each plotted line represents the mean of 3 biological replicates in technical triplicates each. For each gene, dF/dT was normalized by the average peak value of lungs (A-C) or PCCL3 (D-F). NTC: no template control, NAC: no amplification control. Gray: NTC and NAC, black: islets (A-C) or PCCL3 (D-F), red: lungs, blue: kidneys, purple: INS-1E, orange: BRIN-BD11.

Supplementary Figure S4 – Gels in Fig. 3

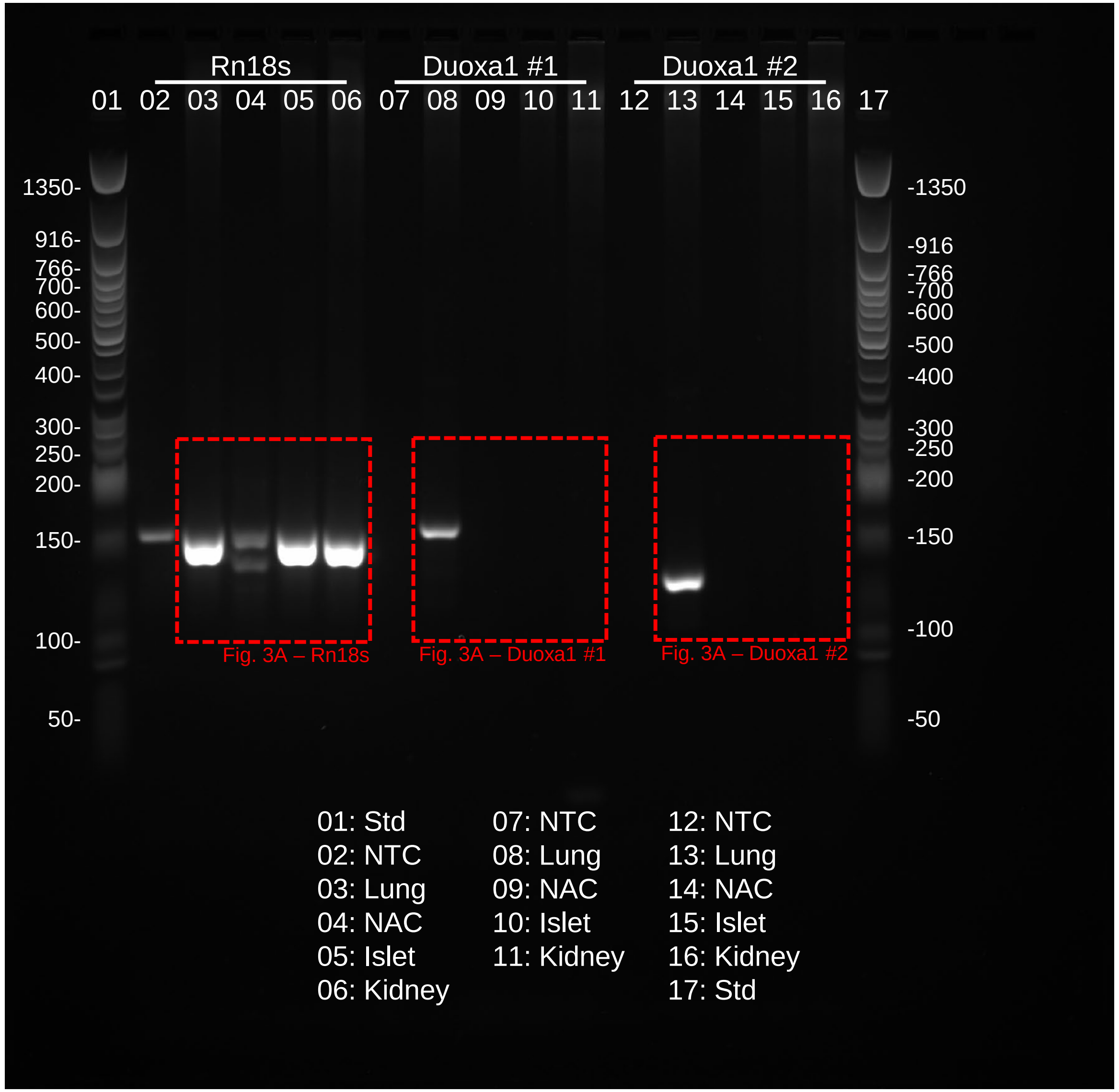

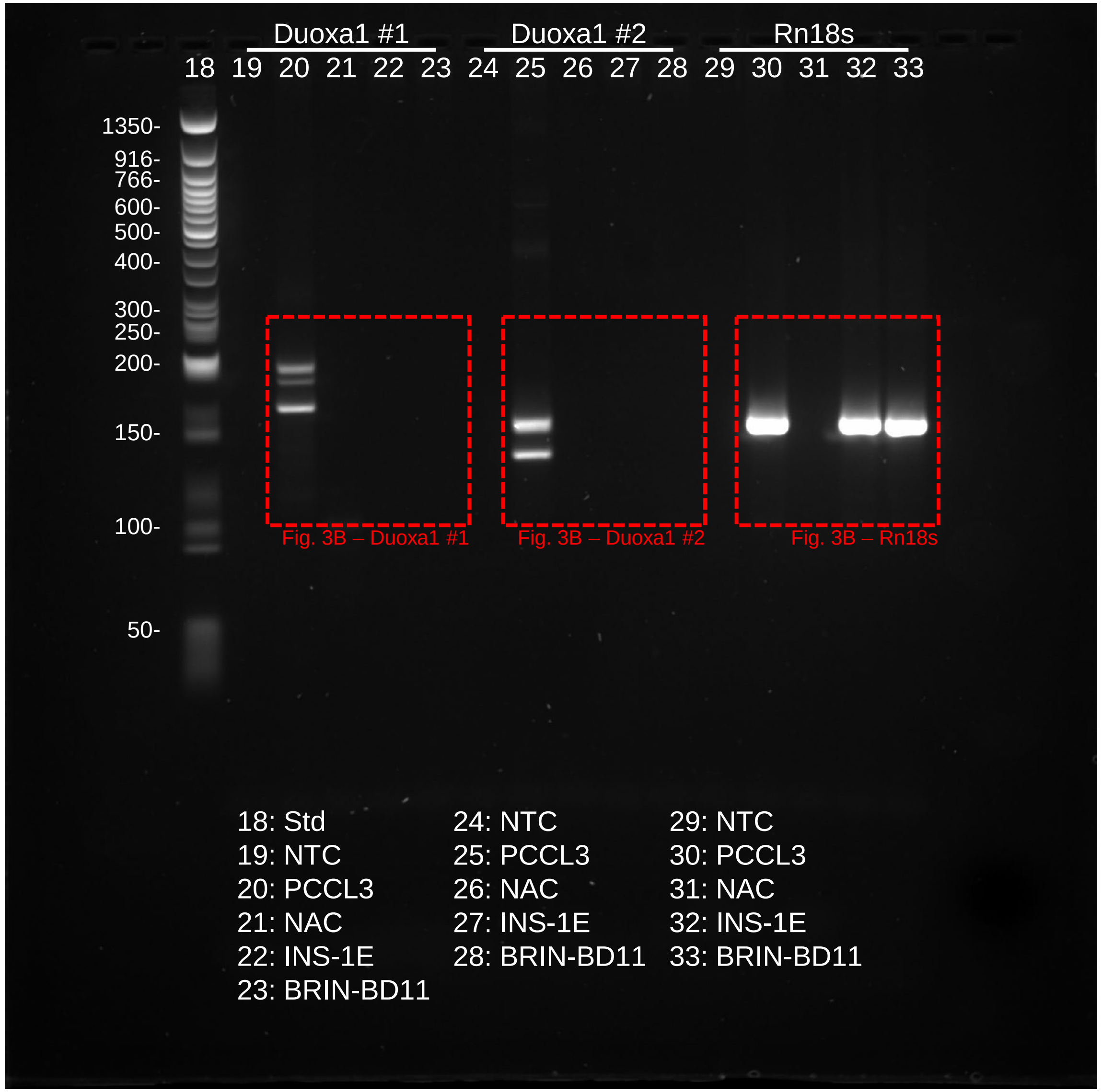

Supplementary Figure S4. Uncropped gels in Fig. 3.

Two uncropped gels are shown. Genes and samples are indicated at the top and the molecular weight in bp to the left. Std: NEB Quick-Load Purple 50 bp DNA Ladder, NTC: no template control, NAC: no amplification control (from Lungs or PCCL3). Red boxes: insets in Fig. 3.

Supplementary Figure S5 – RQ1-3 – Blot 1-2

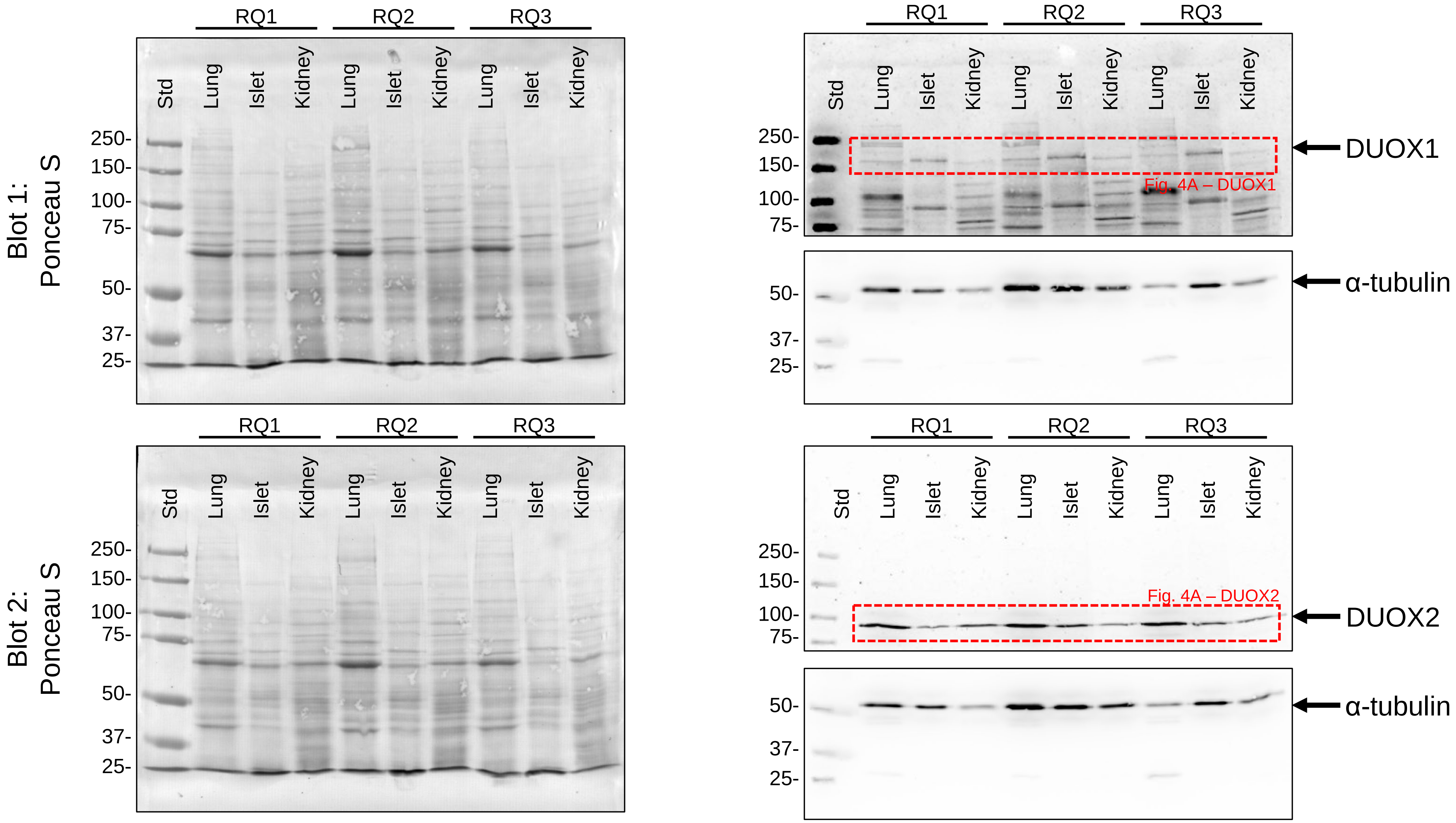

Supplementary Figure S5 – RQ4-5 – Blot 1-2

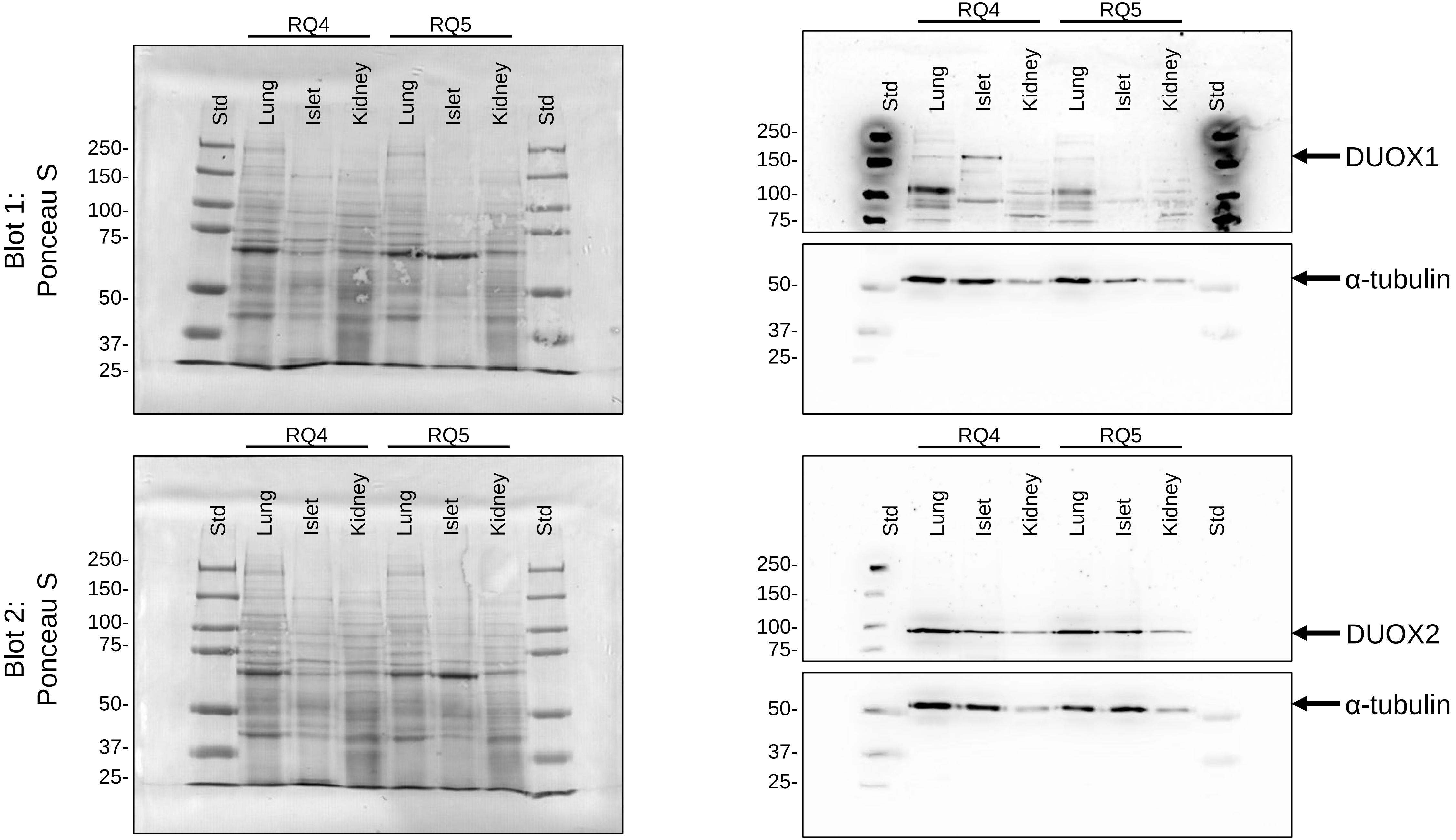

Supplementary Figure S5. Uncropped blots in Fig. 4A.

Four uncropped blots are shown. Ponceau S staining is presented to the left and the respective immunostaining to the right of each slide. Standards were labelled with LI-COR WesternSure Pen. Samples are indicated at the top and the molecular weight in kDa to the left. Std: Bio-Rad Precision Plus Protein Dual Color Standards. Arrows: proteins of interest. Red boxes: insets in Fig. 4A.

Supplementary Figure S6 – IWB – Blot 1-2

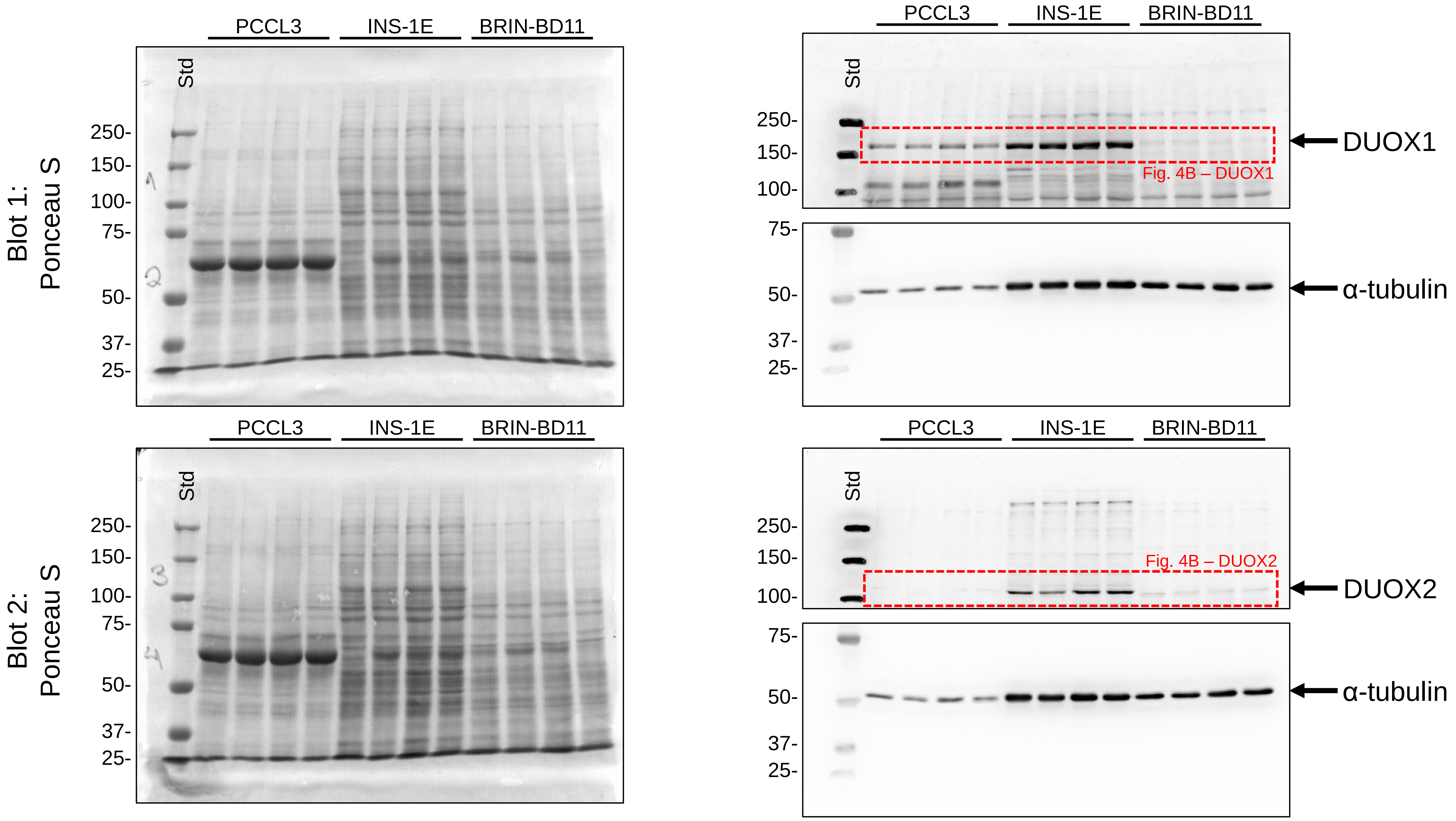

Supplementary Figure S6. Uncropped blots in Fig. 4B.

Two uncropped blots are shown. Ponceau S staining is presented to the left and the respective immunostaining to the right of each slide. Standards were labelled with LI-COR WesternSure Pen. Samples are indicated at the top and the molecular weight in kDa to the left. Std: Bio-Rad Precision Plus Protein Dual Color Standards. Arrows: proteins of interest. Red boxes: insets in Fig. 4B.

Supplementary Figure S7 – CTK9 – Blot 1-2

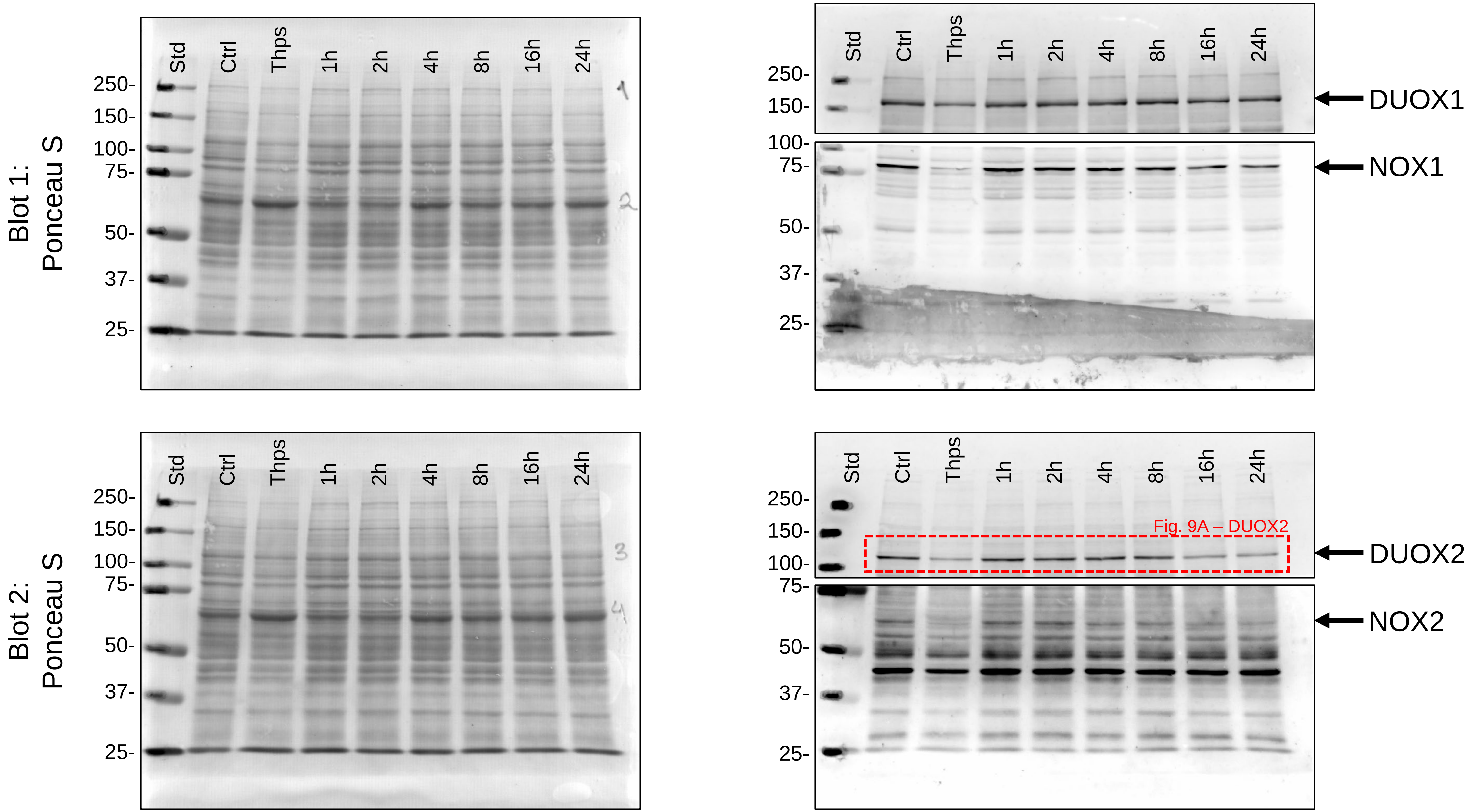

Supplementary Figure S7 – CTK9 – Blot 3-4

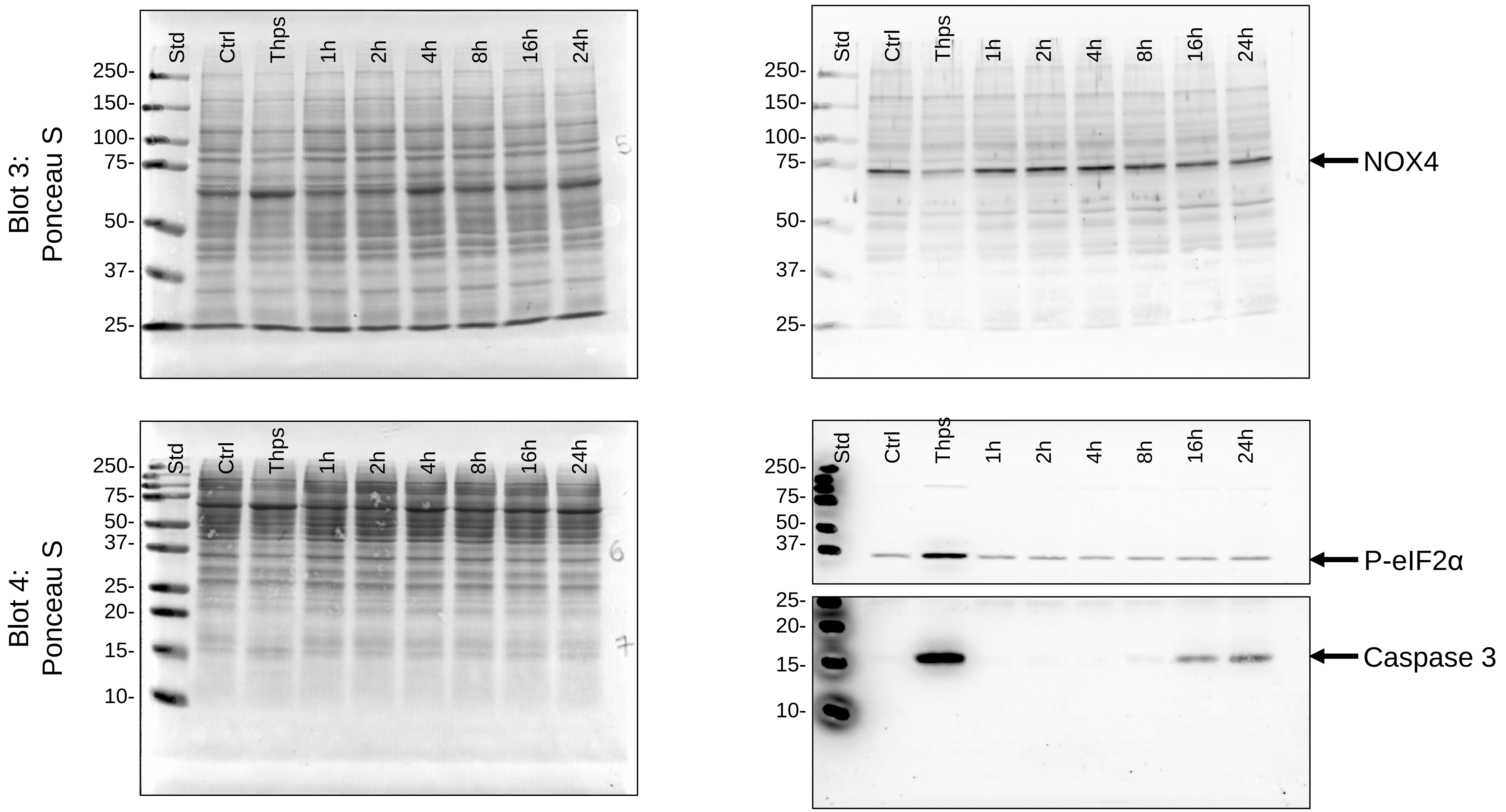

Supplementary Figure S7 – CTK10 – Blot 1-2

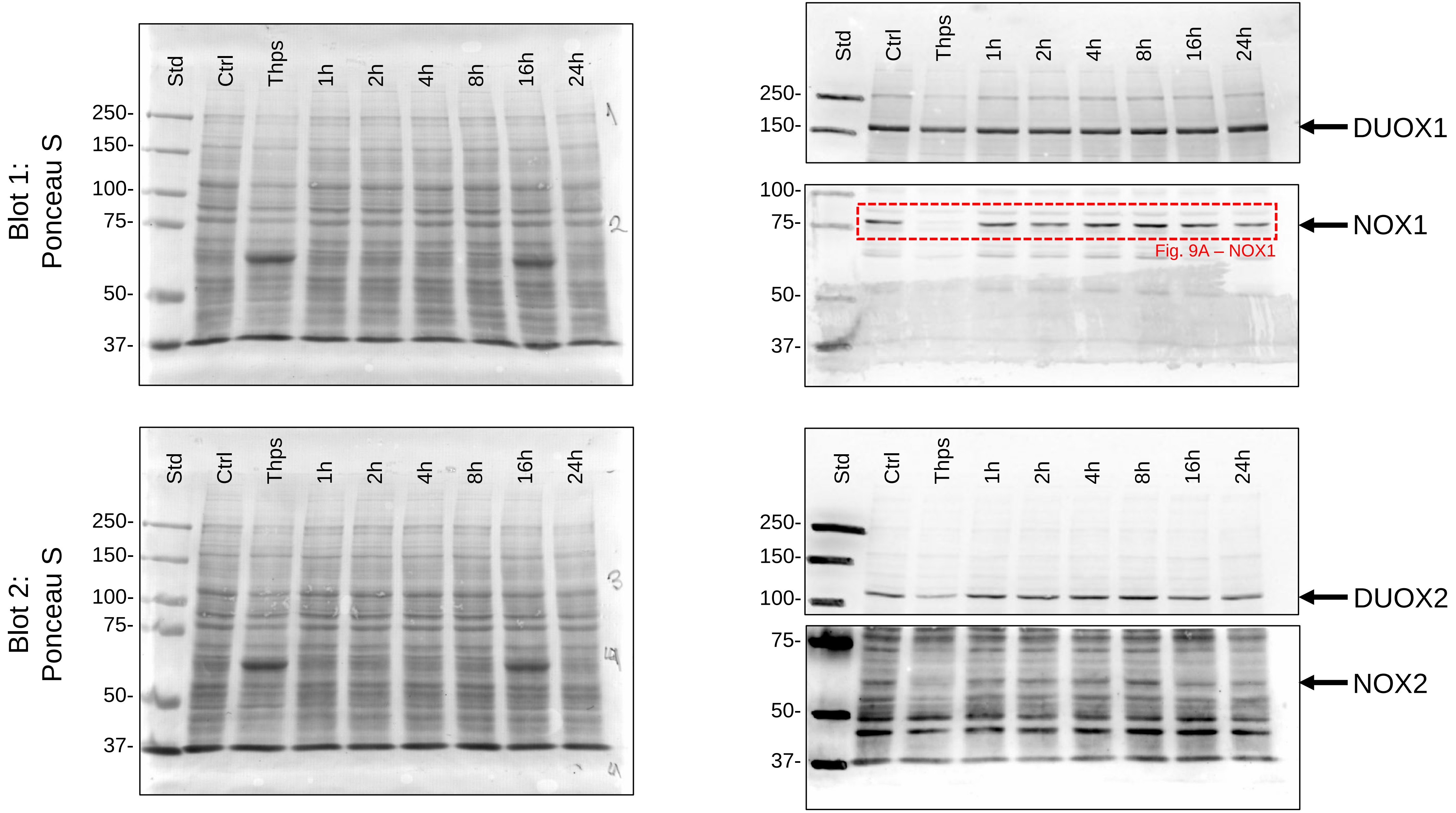

Supplementary Figure S7 – CTK10 – Blot 3-4

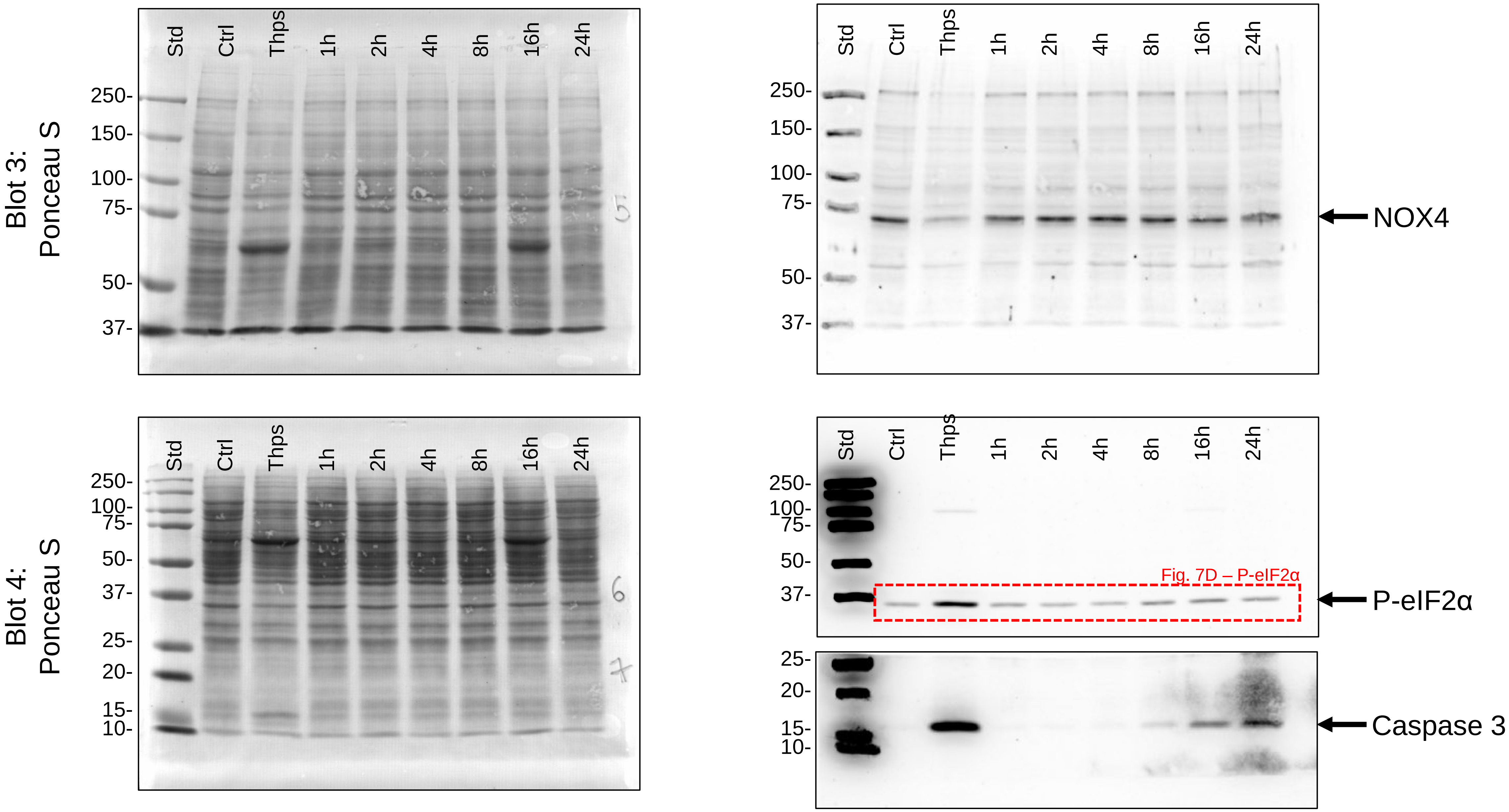

Supplementary Figure S7 – CTK11 – Blot 1-2

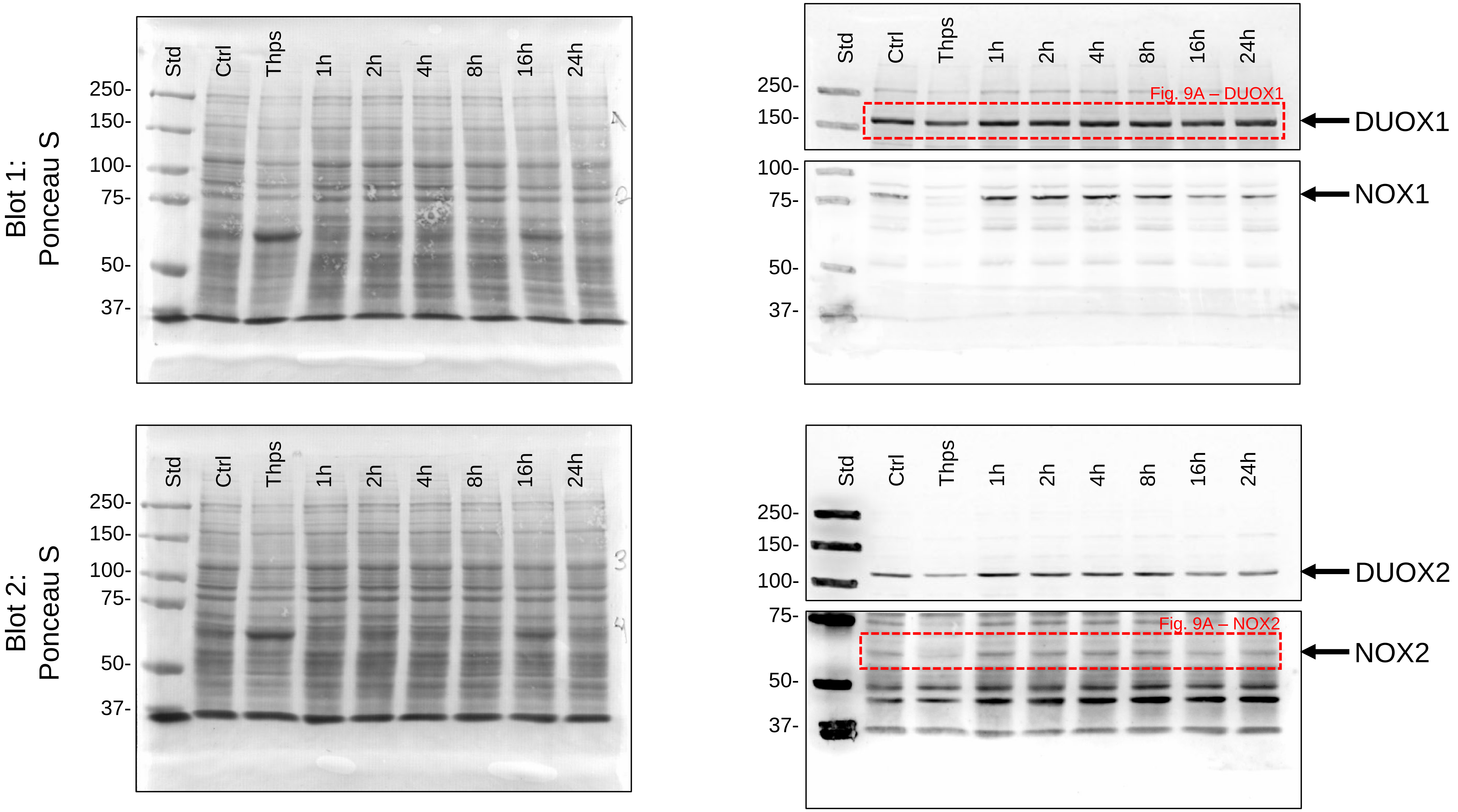

Supplementary Figure S7 – CTK11 – Blot 3-4

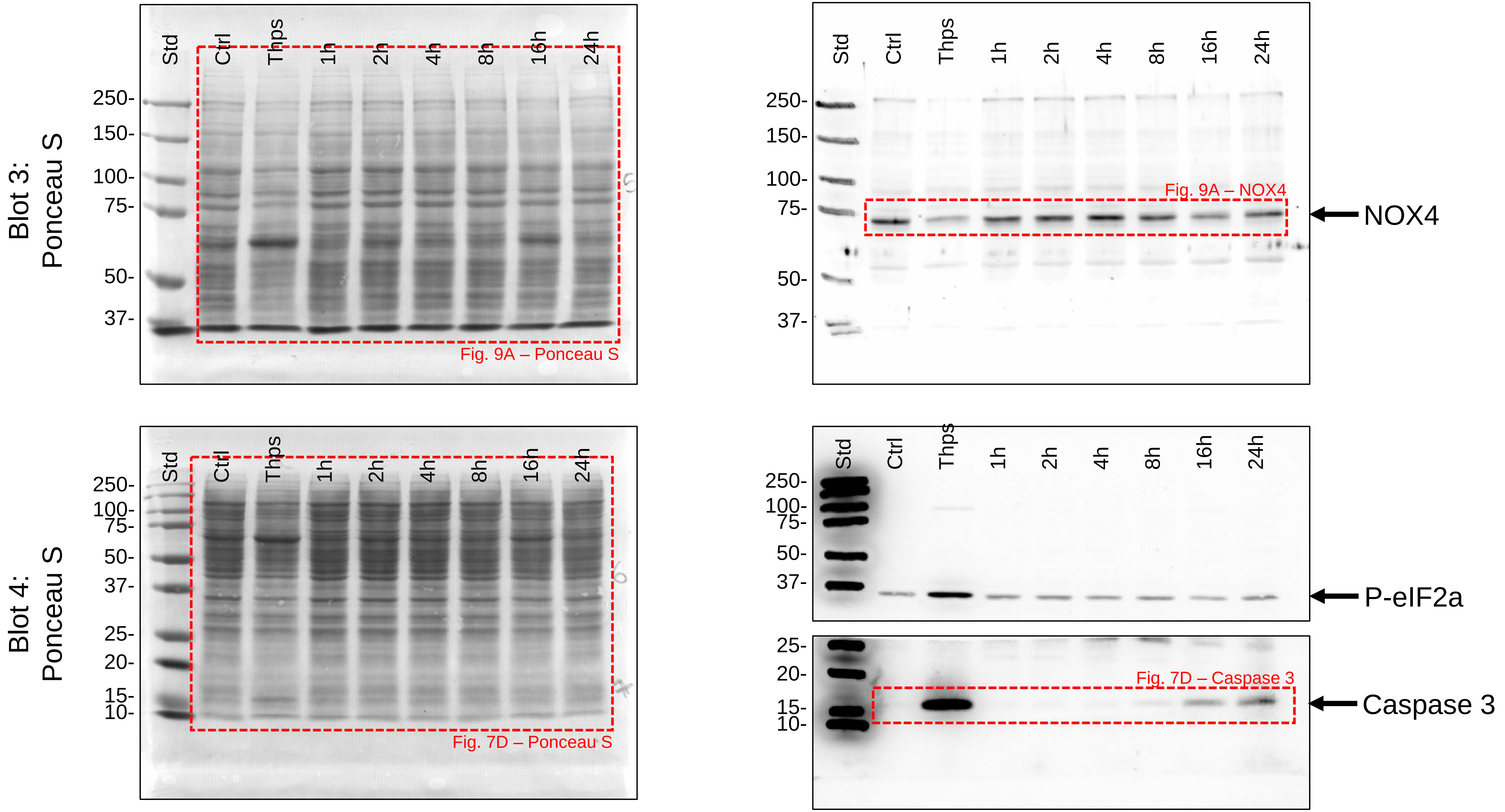

Supplementary Figure S7. Uncropped blots in Fig. 11D and Fig. 13A.

Four uncropped blots per experiment from three experiments (CTK9-11) are shown. Ponceau S staining is presented to the left and the respective immunostaining to the right of each slide. Blots were labelled with pencil and standards labelled with LI-COR WesternSure Pen. Samples are indicated at the top and the molecular weight in kDa to the left. Std: Bio-Rad Precision Plus Protein Dual Color Standards, Ctrl: untreated cells (24 h), Thps: thapsigargin 1 μM (16 h), 1-24 h: cytokine treatment (IL-1β 10 U/mL + IFNγ 14 U/mL + TNF 100 U/mL) for the indicated periods. Arrows: proteins of interest. Red boxes: insets in Fig. 11D and Fig. 13A.
